# β-N-Methylamino-L-Alanine (BMAA) Causes Severe Stress in *Nostoc* sp. PCC 7120 Cells under Diazotrophic Conditions: A Proteomic Study

**DOI:** 10.3390/toxins13050325

**Published:** 2021-04-30

**Authors:** Olga A. Koksharova, Ivan O. Butenko, Olga V. Pobeguts, Nina A. Safronova, Vadim M. Govorun

**Affiliations:** 1 Lomonosov Moscow State University, Belozersky Institute of Physico-Chemical Biology, Leninskie Gory, 1-40, 119991 Moscow, Russia; safronova.na@belozersky.msu.ru; 2 Institute of Molecular Genetics of National Research Center “Kurchatov Institute”, Kurchatov Square, 2, 123182 Moscow, Russia; 3 Scientific-Research Institute of Physical-Chemical Medicine, 119435 Moscow, Russia; ivan.butenko@gmail.com (I.O.B.); nikitishena@mail.ru (O.V.P.); vgovorun@yandex.ru (V.M.G.)

**Keywords:** *Anabaena* sp. PCC 7120, DNA repair, cyanobacteria, inhibitor, nitrogenase, NtcA, oxidative stress response, photosynthesis, nitrogen starvation, cyanotoxin

## Abstract

Non-proteinogenic neurotoxic amino acid β-N-methylamino-L-alanine (BMAA) is synthesized by cyanobacteria, diatoms, and dinoflagellates, and is known to be a causative agent of human neurodegenerative diseases. Different phytoplankton organisms’ ability to synthesize BMAA could indicate the importance of this molecule in the interactions between microalgae in nature. We were interested in the following: what kinds of mechanisms underline BMAA’s action on cyanobacterial cells in different nitrogen supply conditions. Herein, we present a proteomic analysis of filamentous cyanobacteria *Nostoc* sp. PCC 7120 cells that underwent BMAA treatment in diazotrophic conditions. In diazotrophic growth conditions, to survive, cyanobacteria can use only biological nitrogen fixation to obtain nitrogen for life. Note that nitrogen fixation is an energy-consuming process. In total, 1567 different proteins of *Nostoc* sp. PCC 7120 were identified by using LC-MS/MS spectrometry. Among them, 123 proteins belonging to different functional categories were selected—due to their notable expression differences—for further functional analysis and discussion. The presented proteomic data evidences that BMAA treatment leads to very strong (up to 80%) downregulation of α (NifD) and β (NifK) subunits of molybdenum-iron protein, which is known to be a part of nitrogenase. This enzyme is responsible for catalyzing nitrogen fixation. The genes *nifD* and *nifK* are under transcriptional control of a global nitrogen regulator NtcA. In this study, we have found that BMAA impacts in a total of 22 proteins that are under the control of NtcA. Moreover, BMAA downregulates 18 proteins that belong to photosystems I or II and light-harvesting complexes; BMAA treatment under diazotrophic conditions also downregulates five subunits of ATP synthase and enzyme NAD(P)H-quinone oxidoreductase. Therefore, we can conclude that the disbalance in energy and metabolite amounts leads to severe intracellular stress that induces the upregulation of stress-activated proteins, such as starvation-inducible DNA-binding protein, four SOS-response enzymes, and DNA repair enzymes, nine stress-response enzymes, and four proteases. The presented data provide new leads into the ecological impact of BMAA on microalgal communities that can be used in future investigations.

## 1. Introduction

Prokaryotic and eukaryotic phytoplankton species, such as cyanobacteria (cyanoprokaryota), diatoms, and dinoflagellates, synthesize many different secondary metabolites of ecological and practical significance [1,2]. Among them stands out the non-proteinogenic neurotoxic amino acid β-N-methylamino-L-alanine (BMAA), which was related to human neurodegenerative diseases [3,4,5]. The ability of many different microalgae species to synthesize BMAA in a wide range of concentrations—from nanograms to thousands of micrograms per gram of dry weight [6,7,8]—may indicate the biological meaning of this molecule in their life [9,10,11,12]. It was discovered that non-diazotrophic unicellular cyanobacteria start to produce BMAA in nitrogen-limited conditions [13]. Cyanobacteria are not only able to produce BMAA but can also adsorb this amino acid. The cyanobacteria *Synechocystis* PCC 6803 and *Anabaena* (*Nostoc*) PCC 7120 rapidly absorb exogenous BMAA proportionally to the amount of amino acid in the medium [14,15]. Recently, it was proposed that BMAA is taken up mainly through N-I and N-II amino acid transport systems in *Anabaena* sp. PCC 7120 [16].

For the first time, BMAA’s effect on the growth and nitrogenase activity of *Anabaena* (*Nostoc*) PCC 7120 [15] was compared with the effects of 20 standard amino acids and it was shown that BMAA strongly and specifically inhibits cyanobacterial growth and its nitrogenase activity. Later, it was confirmed that BMAA strongly and specifically affects the processes of growth [16,17] and nitrogen fixation [17]. Afterwards the authors experimentally demonstrated by using fluorescent microscopy and transcriptional analysis that BMAA affects cell differentiation in diazotrophic cyanobacteria [17,18] at micromolar concentrations (20–50 µmol). These investigations were performed by using a model cyanobacterial strain *Anabaena* (*Nostoc*) sp. PCC 7120, which is well studied both genetically and physiologically. In botanical literature, this strain is known under the name of *Anabaena* spp. [19,20]. However, during the last decade, this strain has been referred to as *Nostoc* sp. PCC 7120 in the main genomic and protein databases (e.g., https://https.ncbi.nlm.nih.gov/nuccore/BA000019.2 and https://www.uniprot.org/); therefore, further in the text, we will use this name for the mentioned strain.

It should be emphasized that cyanobacteria can face different amounts of nitrogen in their surroundings. In nitrogen-sufficient conditions, filaments of nitrogen-fixing cyanobacteria contain only vegetative cells. Cyanobacteria do not need to waste energy to perform cell differentiation and nitrogen-fixation processes when combined nitrogen sources (nitrate or ammonium) are present in the growth medium. When cyanobacteria are left without enough nitrogen in the medium, they begin to feel “hunger”. They, therefore, develop heterocysts—the nitrogenase-containing specialized cells—to convert atmospheric nitrogen into a more readily assimilable form, such as ammonia [21]. The heterocyst formation process is a short transit period (one–two days) that allows cyanobacteria to grow in a nitrogen-free medium that is more common in nature due to nitrogen-scarcity and competition for nitrogen among microorganisms [22]. 

**Figure 1:**
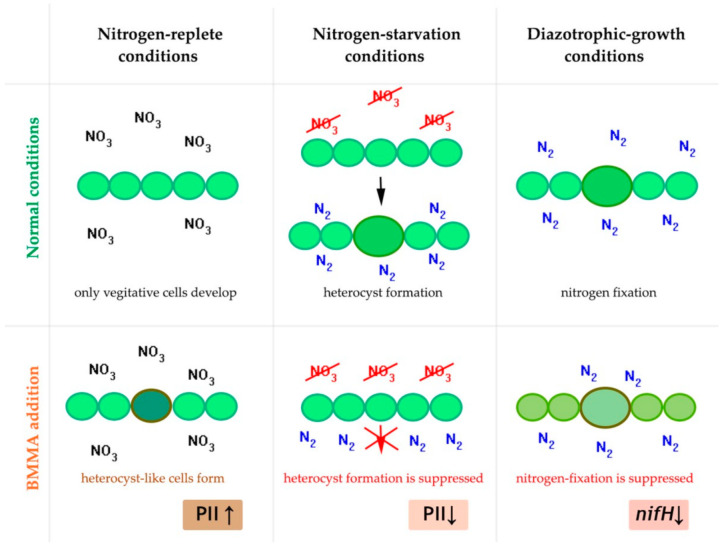
Nitrogen-fixing filamentous cyanobacteria *Nostoc* sp. PCC 7120 under different nitrogen conditions in its normal state and after BMMA treatment. This scheme is based on our results obtained in [17,18,23,24].

In our previous works we have demonstrated by using different experimental approaches that the BMAA’s impact on diazotrophic filamentous cyanobacteria is connected with nitrogen-carbon balance regulation, and is different under nitrogen starvation and in the nitrogen-replete growth medium [17,18,23,24] (Figure 1). The most significant difference in proteome expression consists of regulating a key nitrogen regulatory protein PII under BMAA treatment. This protein is downregulated during nitrogen-starvation, and it is upregulated in nitrogen-rich growth conditions [23,24]. This fact is consistent with the specific regulatory effect of BMAA on heterocyst differentiation and heterocyst- and nitrogenase-related gene expression in *Nostoc* sp. PCC 7120, which was discovered in our previous studies [17,18] by using RT-PCR and microscopy analysis. Since all metabolic processes are well-balanced in cyanobacteria cells, nitrogen metabolism disturbance can lead to changes in carbon metabolism and photosynthesis. That explains the severe downregulation of the expression of CO_2_ fixation proteins and photosystem I (PSI) reaction center proteins found in [23]. BMAA addition leads to disorder in both amino acid synthesis and purine synthesis and disturbs DNA transcription and protein translation. Finally, many oxidative stress enzymes, chaperones, and SOS-response proteins are upregulated under such metabolic stress conditions [23,24].

The proteomic approach can give us a broad picture of the impact BMAA causes on the diazotrophic growth of cyanobacteria in a nitrogen-free medium. To supply all cells in cyanobacterial filaments with nitrogen, cyanobacteria produce mature heterocysts, which are specialized nitrogen-fixing cells that convert atmospheric nitrogen into bioavailable nitrogen and supply with it neighboring vegetative cells. In turn, vegetative cells provide heterocysts with carbon and reductants, which are essentially required for the energy-consuming heterocyst functioning process [21]. Earlier, it was been experimentally shown that BMAA specifically and strongly inhibits the nitrogenase enzyme activity [15,17] and the *nifH* gene expression [17] (Figure 1) in diazotrophically grown *Nostoc* sp. PCC 7120.

This proteomic study aimed to elucidate the effect of exogenous BMAA on the proteome of *Nostoc* sp. PCC 7120 (further referred to as *Nostoc* PCC 7120) in diazotrophic conditions.

## 2. Results and Discussion

### 2.1. Proteins That Are Affected by BMAA under Diazotrophic Conditions

To elucidate the effect of BMAA on *Nostoc* PCC 7120 under diazotrophic conditions cyanobacteria cells were grown in three independent biological replicates in nitrogen-free BG11_0_ medium for 48 h in two experimental settings, in which: (1) control samples consisted of cells grown without BMAA addition and (2) treated samples contained cells grown with BMAA (20 µmol), as it was performed before [23,24]. The analysis of cyanobacteria samples was performed by using the LC-MS/MS method and resulted in identifying 1567 proteins of *Nostoc* PCC 7120 (Supplementary Table S1). Among them, 123 proteins were selected based on the statistical significance of the observed differences between the BMAA-treated samples and control samples for further functional analysis. They were identified as proteins that belong to different functional categories (Table 1, Table 2 and Table 3).

Differently regulated proteins are presented in Table 2 and Table 3. Each table contains the following information: the name of the identified protein, the corresponding gene number, metabolic pathway or function (a possible role of hypothetical proteins), the fold change between BMAA-treated and control samples, and *p*-value. In cyanobacteria cells, BMAA affects proteins with diverse functions within different metabolic pathways. Among them are proteins that are involved in nitrogen fixation, photosynthesis, and oxidative phosphorylation, carbon fixation, carbohydrate metabolism, translation, and transcription, amino acid synthesis, and amino acid metabolism. It was found that in the presence of BMAA, many proteases, stress response, and DNA repair proteins are upregulated (Table 1 and Table 2). Fourteen proteins were shown to be more than two-fold downregulated in BMAA-exposed cells. Twenty-four proteins were more than two-fold upregulated in BMAA-treated cells (Table 1, Table 2 and Table 3). Twenty-six of the identified proteins were specified as “hypothetical” proteins (Table 1 and Table 3). In this study, we found twenty-two proteins encoded by genes, which are under the control of a global transcriptional regulator NtcA [25]. Among them were identified 12 proteins that are downregulated by BMAA and 10 proteins that are upregulated by BMAA (Table 2, Table 3 and Table 4).

The functional category of the selected proteins and their roles in cyanobacteria cells are discussed below in Section 2.2, Section 2.3, Section 2.4, Section 2.5, Section 2.6, Section 2.7, Section 2.8 and Section 2.9.

### 2.2. BMAA Downregulates Nitrogenase Proteins

Diazotrophic cyanobacteria can provide themselves and other organisms that live with them in the same communities with organic nitrogen by using the nitrogenase enzyme complex [26]. Nitrogenase is an enzyme responsible for catalyzing nitrogen fixation, which is a process that reduces atmospheric nitrogen (N_2_) to ammonia (NH_3_). Nitrogen fixation is a necessary process for maintaining life on our planet [27,28]. Nitrogenase can be irreversibly inhibited by molecular oxygen. Cyanobacteria are the only known organisms that can possess active anaerobic nitrogenase and oxygen-producing photosynthesis in one organism by using different oxygen-protective strategies [29,30].

The presented proteomic data show that BMAA treatment leads to strong downregulation of subunits α (NifD) and β (NifK) of nitrogenase molybdenum-iron protein (component I) (Table 2). These two proteins form a network with other proteins that are involved in nitrogen metabolism (Figure S1). Among them a glutamine synthetase (glnA), glutamate dehydrogenase (alr4255), and nitrite reductase (nirA) draw attention. Glutamine synthetase (glnA) is involved in ammonium assimilation. GlnA catalyzes the ATP-dependent biosynthesis of glutamine from glutamate and ammonia. Glutamate dehydrogenase (alr4255) and nitrite reductase (nirA) are also involved in nitrogen assimilation.

Note that the corresponding genes (*nifD* and *nifK*) are under the transcriptional control of NtcA [25]. This effect of BMAA on nitrogenase proteins can explain the data obtained earlier in [15,17], which shows a strong BMAA-specific inhibitory effect on nitrogenase activity. It is known that nitrogenase activity is sensitive to oxygen, which inactivates this enzyme [31]. Moreover, nitrogenase activity can be inactivated by nitrate and ammonium [32,33], and other nitrogen-containing sources [15,34,35]. Therefore, we can assume that different mechanisms can explain BMAA’s inhibition of nitrogenase. The reaction of BMAA with pyridoxal-5′-phosphate resulted in the production of methylamine and ammonia as final products. Afterwards, the methylamine was oxidized into formaldehyde, hydrogen peroxide, and ammonia [36,37,38], which can inhibit nitrogenase.

Another mechanism of BMAA action on prokaryotic and eukaryotic cells lies in its influence on the synthesis and degradation of glutamate and glutamine. For example, it has been experimentally shown that BMAA inhibits the synthesis and/or stimulates glutamine degradation in rat tissues [36]. Additionally, BMAA induces glutamate the loss of by affecting the antiport system cystine/glutamate in mouse cell cultures [39]. In this regard, it has been suggested that amino acid BMAA inhibits nitrogenase activity in cyanobacteria not due to being a potential source of nitrogen, but through a mechanism that affects glutamate and glutamine metabolism. Glutamate is an acceptor of ammonium ions, which are produced by nitrogenase in cyanobacteria heterocysts, where glutamine is synthesized and afterwards exported to vegetative cells [21]. Glutamine serves as a precursor to glutamate and eventually to all other amino acids in cyanobacteria and plant cells. It can be assumed that exposure to BMAA reduces the level of glutamine and stimulates the release of glutamate from cyanobacterial cells (this fact was observed in mouse cells [39]). Therefore, BMAA presence could lead to a rapid intracellular accumulation of NH_4_^+^ in cyanobacteria that is followed by the inhibition of nitrogen fixation. It is well known that BMAA inhibits nitrogen fixation in *Nostoc* PCC 7120 [15,17]. Moreover, it appears from the data obtained using isotope-labeled BMAA (L-BMAA-4,4,4-D3, ^15^N_2_), that the primary amino group of BMAA is transferred to other amino acids in *Synechocystis* PCC 6803 cells [10]. In particular, the ^15^N_2_-label from BMAA was redistributed between free glutamine and glutamic amino acids. It was found that the redistribution of the ^15^N_2_-label from BMAA to glutamate is blocked in the case of glutamate synthase inhibition. According to the authors [10], this may be an indicator of this enzyme’s participation in BMAA metabolism inside of cyanobacterial cells. The possible relationship between BMAA synthesis in cyanobacterial cells and nitrogen metabolism was recently considered in a preliminary study [40].

Both described mechanisms can explain BMAA’s impact on nitrogen fixation. It was demonstrated in two previous proteomic studies [23,24] and in the present study (Table 2, Section 2.6) that BMAA affects enzymes involved in glutamate metabolism. At the same time, the discussed above reaction of BMAA with pyridoxal-5′-phosphate can occur, leading to oxidative stress, which can trigger diverse defense mechanisms. For example, BMAA treatment can lead to the upregulation of many stress-response enzymes (Section 2.7).

In the current proteomic study, it was found that, under BMAA treatment, the DNA binding protein Abp2 (*all1939*) [41] was upregulated (Table 2). This protein is a transcription factor, which is essential for the expression of *hepC* and *hepA* genes and is important for the subsequent heterocyst maturation in *Nostoc* (*Anabaena*) PCC 7120 [41]. As it was demonstrated earlier, Abp2 mutation leads to the complete inactivation of *hepC* and *hepA* gene expression and, therefore, prevents both heterocyst maturation and aerobic nitrogen fixation. As shown in [41] using thin-layer chromatography of lipids and transmission electron microscopy, Abp2 mutant does not form heterocyst envelope glycolipids. Hence, it cannot fix nitrogen in an oxygen-containing milieu [41]. Protein Abp2 is downregulated when heterocyst formation is blocked under BMAA treatment [23]. In this study, it was found that the Abp2 protein is upregulated under BMAA exposure. Thus, we can suppose that more heterocyst glycolipids are probably needed to protect the downregulated nitrogenase from oxygen in the presence of BMAA.

### 2.3. BMAA Downregulates Photosynthesis and Oxidative Phosphorylation Proteins

**Figure 2:**
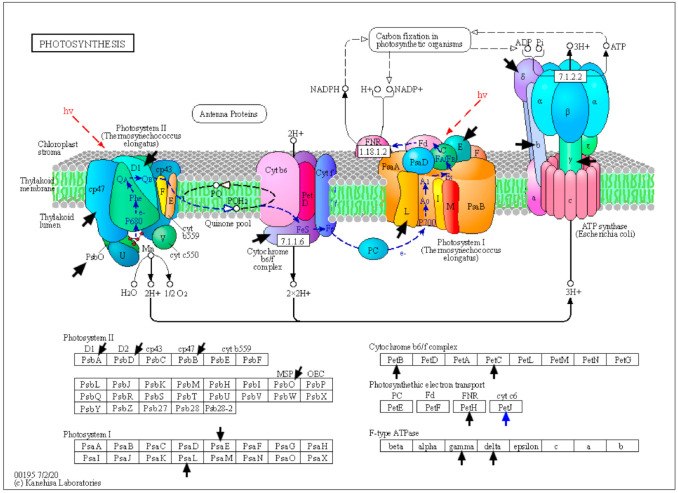
The scheme represents photosynthetic protein complexes (https://www.genome.jp/kegg-bin/show_pathway?ana00195+asr4319) and the effect BMAA causes on the protein components of photosystem I, photosystem II, cytochrome b6/f complex, and ATP synthase that were found in this study. Black arrows point at downregulated proteins and one blue arrow points at the only upregulated protein Cyt A (cytochrome c6) under BMAA treatment.

Cyanobacteria perform oxygenic photosynthesis by using water as a source of electrons that are transferred from water to CO2 in order to reduce CO2 to various organic compounds. In these organisms, the linear electron transfer takes place in the thylakoid membranes due to the activity of photosystem II (PSII), cytochrome b6f (Cytb6f), and photosystem I (PSI) (Figure 2). These multi-protein complexes transform solar energy and, together with ATP synthase, create reducing power (NADPH) and chemical energy (ATP) that are used to produce carbohydrates in the Calvin cycle [42]. The present proteomic analysis has revealed that BMAA affected 19 proteins involved in photosynthesis and 6 proteins that participate in oxidative phosphorylation (Table 2, Figure 2). It was found that BMAA downregulates 18 photosynthesis-associated proteins and all six proteins involved in oxidative phosphorylation (five subunits of ATP synthase F0F1 and the subunit H of NAD(P)H-quinone oxidoreductase) (Table 2, Figure 2).

In cyanobacteria cells, all metabolic processes are well co-regulated and balanced. Such coordinated regulation allows cyanobacteria to adapt to continually changing growth conditions, where the availability of nitrogen, carbon, and other elements, and lighting conditions are not constant. Alterations in nitrogen supply lead to many changes in carbon fixation and photosynthesis regulation [43,44]. A global regulatory transcription factor NtcA acts as a transcriptional activator or repressor. It directly regulates the expression of multiple genes required for nitrogen and carbon assimilation, photosynthesis, DNA metabolism, transcription and translation, and other processes [25,44,45]. In our study, several proteins that are encoded by NtcA-regulated genes were found to be downregulated in the presence of BMAA in diazotrophic growth conditions. Among them, there are two nitrogenase proteins (NifK and NifD) and two photosynthetic proteins (psbA1 and psbD), as well as four proteins, which are involved in oxidative phosphorylation (atpC,D,F and ATP synthase F0F1 subunit A) (Table 2).

BMAA addition leads to the downregulation of two proteins of the PSI reaction center (subunits IV and XI). Besides that, BMAA downregulates four proteins of PSII (D1,D2,CP47, psbO) and five proteins that are components of the phycobilisome light-harvesting antennae of photosystem II (Table 2, Figure 2). Additionally, four enzymes involved in the porphyrin and chlorophyll metabolism were also downregulated (Table 2, Supplementary Figure S2). Among the downregulated proteins were identified ferredoxin-NADP(+) reductase (petH) and two subunits of the cytochrome b6-f complex (petB and petC) (Table 2, Figure 2).

Only one protein, cytochrome c6 (*alr4251*), was found upregulated in BMAA treated cells (Table 2). Cytochrome (Cyt) c6 transfers electrons between the Cytb6-f complex and photosystem I (PSI) in the thylakoidal lumen of cyanobacteria and green algae [46]. It was shown that this protein is the main respiratory and photosynthetic soluble electron donor in heterocysts of *Anabaena* sp. PCC 7120 [47]. The upregulation of Cyt c6 under BMAA treatment could be considered as a kind of compensation event in the respiratory electron transport and photosynthetic electron transport while several other photosynthetic proteins were downregulated.

### 2.4. BMAA Impact on the CO_2_ Concentrating Mechanism and Bicarbonate Transport

Cyanobacteria possess a single-cell CO_2_ concentrating mechanism (CCM) that allows them to increase the concentration of CO_2_ at the site of ribulose-1,5-bisphosphate carboxylase/oxygenase (Rubisco), which is known as a primary CO_2_-fixing enzyme [48,49,50]. This mechanism helps cyanobacteria to actively concentrate dissolved inorganic carbon into their cells and adapt to various environmental limitations. The CCM consists of two functional elements. The first one is associated with carboxysomes and their protein microbodies that are the cell compartments for Rubisco (the sites for CO_2_ elevation), and the second functional element is related to several inorganic carbon (Ci) transporters that deliver HCO_3_^−^ into cells [51,52]. One of them is the complex that is composed of two ATP-binding proteins (CmpC and CmpD), a transmembrane protein (CmpB), and a solute-binding protein (CmpA) [53]. Our proteomic study found that bicarbonate transport ATP-binding protein CmpD is strongly downregulated in BMAA treated cells of *Nostoc* PCC 7120 (Table 2). This protein has been identified only in control samples. Figure S3 (Supplementary Figure S3) shows the protein network of CmpD and its protein partners. Among them are CmpA, CmpB, and CmpC, which are different parts of the ABC transporter complex CmpABCD, which is involved in bicarbonate transport. In cases of protein CpmD absence, the whole complex has to become nonfunctional. Moreover, CmpD interacts with other proteins, such as nitrate transport proteins NrtA and NrtB; therefore, the absence of CmpD protein ought to affect nitrate transporters’ functionality.

### 2.5. Changes in Carbohydrate Metabolism Proteins’ Regulation Caused by BMAA in Diazotrophic Conditions

The inhibitory effects BMAA causes on photosynthesis, oxidative phosphorylation, the CO_2_ concentrating mechanism, and bicarbonate transport lead to changes in the regulation of carbohydrate anabolic and catabolic processes (Table 1 and Table 2). We have found 14 enzymes that were affected by the presence of BMAA. Six proteins were downregulated, and eight proteins were upregulated.

Four enzymes of the anabolic pentose phosphate pathway were downregulated under BMAA treatment (Supplementary Figure S4). Among them were found the following proteins: 6-phosphogluconate dehydrogenase (*alr5275*) [EC:1.1.1.44 1.1.1.343]; transketolase (*alr3344*) [EC:2.2.1.1]; fructose-bisphosphate aldolase, class II, (*all4563*) [EC:4.1.2.13], and fructose-1,6-bisphosphatase II/sedoheptulose-1,7-bisphosphatase (FBP/SBPase) (*alr1041*) [EC:3.1.3.11 3.1.3.37]. The last two enzymes are known to also function in gluconeogenesis in cyanobacteria. Both of them were downregulated at least two-fold (Table 2, Figure S5). Enzyme FBP/SBPase is unique in the way it catalyzes two separate reactions in the Calvin cycle, both of which are catalyzed by distinct enzymes in plants [54]. It is known that cyanobacteria have two FBPase isozymes that are designated as FBPase-I and FBPase-II; the first one belongs to a new type of FBPase and can hydrolyze both fructose 1,6-bisphosphate (Fru 1.6-P2) and sedoheptulose 1,7-bisphosphate (Sed 1,7-P2), while the second one is a typical enzyme that is similar to the enzymes that are present as cytosolic and chloroplastic forms in eukaryotic cells [55,56,57]. In our study, we have found that the two enzymes FBPase-I (*all4021*) and FBPase-II (*alr1041*), are regulated differently in the presence of BMAA (Table 2). Fructose-1,6-bisphosphatase I (*all4021*) [EC:3.1.3.11] was found only in BMAA treated samples, while fructose-1,6-bisphosphatase II/sedoheptulose-1,7-bisphosphatase (*alr1041*) [EC:3.1.3.11 3.1.3.37] was downregulated under BMAA treatment.

Two of the downregulated anabolic enzymes, the 6-phosphogluconate dehydrogenase [EC:1.1.1.44 1.1.1.343] and the fructose-bisphosphate aldolase, class II [EC:4.1.2.13], are under NtcA control.

Seven enzymes involved in the catabolic processes (in glycolysis and starch and sucrose metabolism) were upregulated in the presence of BMAA (Table 2). Two enzymes (glycogen phosphorylase [EC:2.4.1.1] and glgB 1,4-alpha-glucan branching enzyme [EC:2.4.1.18]), which are involved in glycogen catabolism, were upregulated. Glycogen phosphorylase was upregulated more than two-fold. This enzyme breaks up glycogen into glucose subunits under starvation conditions [58]. The 2.5-fold upregulated enzyme, rfbB, UDP-glucuronate decarboxylase [EC:4.1.1.35], participates in starch and sucrose metabolism and nucleotide sugar metabolism. This enzyme has one substrate, UDP-D-glucuronate, and two products, UDP-D-xylose and CO_2_ [59,60].

Summarizing the facts mentioned above we can state that in the conditions of substrate and energy limitations, anabolic processes are downshifted in cyanobacteria cells due to suppression of the following processes: nitrogen fixation, carbon transport, photosynthesis, and the decline of energy supply. Starving cyanobacteria cells have no other choice but to use their early accumulated internal carbon resources and therefore enhance the catabolic processes.

### 2.6. Amino Acid Biosynthesis, Metabolism, and Transport

One enzyme was downregulated, and ten enzymes involved in amino acid metabolism have been found upregulated in the presence of BMAA in cyanobacterial cells, which were grown in diazotrophic conditions (Table 2).

Urease subunit α (*alr3670*) [EC:3.5.1.5] is downregulated in the way it changes in nitrogen starvation growth conditions [23]. However, in nitrogen-replete conditions, this subunit is upregulated under BMAA treatment [24]. Urease subunit α is the central functional part of the nickel-containing metalloenzyme that catalyzes the hydrolysis of urea into carbon dioxide and ammonia [61]. Urease participates in arginine and purine metabolism. It is a widespread enzyme in cyanobacteria, and most cyanobacteria possess genes encoding urease [62]. Earlier, the previous studies demonstrated that protein synthesis inhibition leads to the degradation of urease in cyanobacteria [63]. Later on, it was shown that ammonium promotes urease synthesis repression and has to be metabolized by glutamine synthetase before repressing the urease activity [64]. Therefore, you can expect that the urease subunit α should be downregulated due to the changes in the regulation of the proteins involved in translation (Section 2.7) and glutamate-glutamine metabolic processes in limited nitrogen supply conditions under BMAA treatment (Table 2).

Among the ten upregulated amino acid metabolic enzymes, five enzymes are involved in alanine, aspartate, and glutamate metabolism (Table 2, Figure S6). They are known as the following: carbamoyl-phosphate synthase large subunit [EC:6.3.5.5], adenylosuccinate synthase [EC:6.3.4.4], succinate-semialdehyde dehydrogenase/glutarate-semialdehyde dehydrogenase [EC:1.2.1.16 1.2.1.79 1.2.1.20], aspartate aminotransferase [EC:2.6.1.1] and glutamine-fructose-6-phosphate transaminase (isomerizing), nodM, [EC:2.6.1.16]. The last enzyme, NodM (*alr3464*), was upregulated in *Nostoc* PCC 7120 under BMAA treatment in all three different growth conditions, i.e., during nitrogen starvation [23], in the nitrogen-replete medium [24], and during diazotrophic growth (Table 2). This enzyme participates in glutamate metabolism and amino sugars metabolism. It performs a reaction in which the two substrates of this enzyme, L-glutamine and D-fructose 6-phosphate, are converted into two products—L-glutamate and D-glucosamine 6-phosphate.

As it has been analyzed and discussed before [23], NodM participates in the GlnA and GlnB (PII) protein network. We have also pointed out that BMAA, presumably by acting as a glutamate analog (for review, see [11,12]), can change glutamate metabolism enzymes’ regulation. In this study, NodM was found to be upregulated almost two-fold. The upregulation of no less than five glutamate synthesis enzymes (Table 2) permits us to assume that cyanobacterial cells require glutamate in the presence of BMAA. Moreover, as has been discussed in Section 2.2, a strong downregulation (five-fold change) of the NifK protein occurs under BMAA treatment. This downregulation of nifK must have an impact on its protein partners’ functioning (Figure S1). Among nifK protein partners, there is glutamine synthetase (GlnA), an enzyme that is directly involved in glutamate and glutamine metabolism. The downregulation of the key nitrogenase proteins (Table 2) and, accordingly, the downregulation of nitrogenase enzyme activity caused by BMAA [15,17], leads to blockage of the process of ammonium incorporation into carbon skeletons that is performed by the sequential action of glutamine synthetase (GS) and glutamate synthase (GOGAT).

Among the found upregulated enzymes involved in amino acid metabolism, four enzymes are under a global transcription regulator NtcA control (Table 2). Two of them are involved in glutamate synthesis (carbamoyl-phosphate synthase large subunit [EC:6.3.5.5] and aspartate aminotransferase [EC:2.6.1.1]) and two others, valine-pyruvate aminotransferase [EC:2.6.1.66] and threonine synthase [EC:4.2.3.1], participate in valine, leucine, and isoleucine biosynthesis and glycine, serine, and threonine metabolism, correspondingly. Moreover, the upregulated periplasmic amino acid-binding protein, ABC transporter (*alr4164*) (Table 2), is also known to be under NtcA control. This protein is involved in the transport of amino acids Asp, Glu, Asn, Gln, and Met [65], and is interconnected with other proteins that are involved in amino acid transport (Supplementary Figure S7).

### 2.7. Transcription and Translation

BMAA affects proteins that are involved in DNA transcription and protein synthesis (Table 2). Two proteins involved in transcription—DNA-directed RNA polymerase, subunit α [EC:2.7.7.6], and anti-termination protein NusA—are upregulated at BMAA presence. Before, we have found out that two subunits of DNA-directed RNA polymerase (rpoB and rpoC1) were upregulated in *Nostoc* PCC 7120 in the presence of BMAA during nitrogen starvation [23]. In this study, subunit α (rpoA) was upregulated (Table 2). Such upregulation of subunits of DNA-directed RNA polymerase may be explained by severe stress triggered by BMAA in nitrogen limitation conditions. It was demonstrated that the expression level of plant genes encoding rpoB and rpoC1 is induced by various stresses [66].

Another protein, transcription elongation factor NusA, is a multidomain regulator of transcript elongation is almost two-fold upregulated under BMAA treatment (Table 2). The NusA protein interacts with elongating complexes and the nascent RNA transcript in ways that stimulate pausing and termination. Still, it can be switched to anti-pausing and anti-termination by other accessory proteins [67]. This protein is also involved in DNA repair [68]. Upregulation of this protein well agrees with the upregulation of four DNA repair enzymes (Section 2.8).

The translation process was also affected by BMAA, as it was found before in the two first proteomic studies [23,24]. In diazotrophic conditions, eight proteins of *Nostoc* PCC 7120, involved in protein synthesis, were differently regulated at the BMAA presence. Two of them, 30S ribosomal protein S7 and 50S ribosomal protein L19, were remarkably downregulated as well as RNA-binding protein rbpD (Table 2). The small subunit ribosomal protein S7 was downregulated in *Nostoc* PCC 7120 cells during nitrogen starvation at BMAA presence [23].

RNA-binding protein rbpD (*asl4022*) was downregulated more than three-fold (Table 2) in diazotrophically grown *Nostoc* PCC 7120 cells under BMAA treatment. Note that this protein was oppositely regulated in nitrogen-replete cells. It was found almost three-fold upregulated in BMAA presence [24]. This protein belongs to cyanobacterial stress-inducible RNA-binding proteins (Rbps) [69], and it was found that nitrogen nutrition modulates the stress-responsive regulation of RNA-binding proteins concerning cold stress and then in response to osmotic stresses [70]. In the proteomic study [71], RbpD protein was downregulated in the deletion mutant (AnΔahpC) of *Anabaena* sp. PCC 7120 with impaired protein alkylhydroperoxide reductase (AhpC), the antioxidant protein that is a unique member of the peroxiredoxin family [71].

According to the STRING database (https://string-db.org), RNA-binding protein rbpD protein (*asl4022*) interconnects with its protein partners (Figure S8). Among them is the gltX, Glutamate-tRNA ligase, which catalyzes the attachment of glutamate to tRNA(Glu). This enzyme was found absent at BMAA treatment of nitrogen starving *Nostoc* PCC 7120 cells in our previous proteomic study [23]. The other protein partner of RbpD protein is NtcB, a Nitrogen assimilation transcriptional activator (*all0602*) [45,72]. The third protein partner of rbpD protein is the enzyme of glycolysis, the 2,3-bisphosphoglycerate-independent phosphoglycerate mutase, gpml (*all4182*). This protein catalyzes the inter-conversion of 2-phosphoglycerate and 3-phosphoglycerate. It could be proposed that three-fold downregulation of rbpD protein may affect functional relations with its protein partners.

Two other enzymes involved in translation—glyS, glycyl-tRNA synthetase beta chain [EC:6.1.1.14] and aspS, aspartyl-tRNA synthetase [EC:6.1.1.12]—were found upregulated. In contrast, the phenylalanyl-tRNA synthetase β chain [EC:6.1.1.20] was slightly downregulated at BMAA presence (Table 2). As it was reviewed previously [11], the biological impact of BMAA on living organisms is pleiotropic and can involve different mechanisms. One such mechanism is a disturbance of protein synthesis by BMAA. For example, in human cells, BMAA is mistakenly incorporated into proteins instead of L-serine [73]. It was recently shown that BMAA is a substrate for human alanyl-tRNA synthetase (AlaRS) [74]. It forms BMAA-tRNAAla by escaping from the intrinsic AlaRS proofreading [74]. In the same study, the authors mentioned that cyanobacterial AlaRS also activates BMAA.

### 2.8. Proteases, Stress Response Proteins, and DNA Repair Enzymes Are Significantly Upregulated at BMAA Presence

The BMAA treatment leads to a disturbance in the regulation of many proteins involved in nitrogen metabolism, carbon dioxide concentration mechanism, photosynthesis, amino acid metabolism, protein synthesis, and DNA transcription in cyanobacteria cells. The apparent imbalance in the cell metabolism between different metabolic processes leads to strong intracellular stress. Such internal stress was evidenced by the upregulation of four proteases, two chaperone proteins, four DNA repair enzymes, and nine stress response proteins.

Three protease proteolytic subunits of the ATP-dependent Clp protease [EC:3.4.21.92] were upshifted during the diazotrophic growth of *Nostoc* PCC 7120 under BMAA treatment (Table 2). ATP-dependent Clp protease plays a significant role in the degradation of misfolded proteins. It is known as a protein composed of subunits with a degradative activity against short peptides, which undergo cleavage in various proteins in an ATP-hydrolysis-dependent process [75]. Another protease, the carboxyl-terminal processing protease [EC:3.4.21.102], was upregulated as well (Table 2). This protease cleaves peptide bonds at the C-terminal end (C-terminus) of polypeptides and is primarily involved in posttranslational protein processing, protein maturation, or degradation [76]. Carboxyl-terminal processing protease is an essential peptidase that is crucial for the functionality of the PS II reaction center in cyanobacteria and higher plants [77,78]. This enzyme cleaves a precursor of D1 protein (a key subunit of the PS II reaction center) at the C-terminal extension, which typically consists of 9–16 amino acid residues [76]. Note also that one protease was downregulated in the presence of BMAA. Serine proteinase (*alr2758*) was 1,5-fold downregulated at BMAA presence (Table 2). In prokaryotes, trypsin-like serine proteases function in diverse processes [79]. The revelation of the concrete role of serine proteinase (*alr275*) in *Nostoc* PCC 7120 and the reason behind its downregulation caused by BMAA in diazotrophic conditions are interesting subjects for upcoming genetic and biochemical studies.

BMAA treatment induced nine enzymes involved in stress response (Table 2). These data are supported by the data obtained previously in studies of eukaryotic and prokaryotic (cyanobacteria) cells, which have demonstrated that oxidative stress induction occurs due to the addition of BMAA [12,23,24,39]. In nature, cyanobacteria are often exposed to changing external conditions, such as rapid and drastic fluctuations of light intensity, nutrient availability changes, and toxins’ appearance. Therefore, they have developed an amazing ability to initiate antioxidant defense rapidly [80,81,82]. In the present proteomic study, a severe BMAA impact on *Nostoc* PCC 7120 proteins was detected.

**Figure 3:**
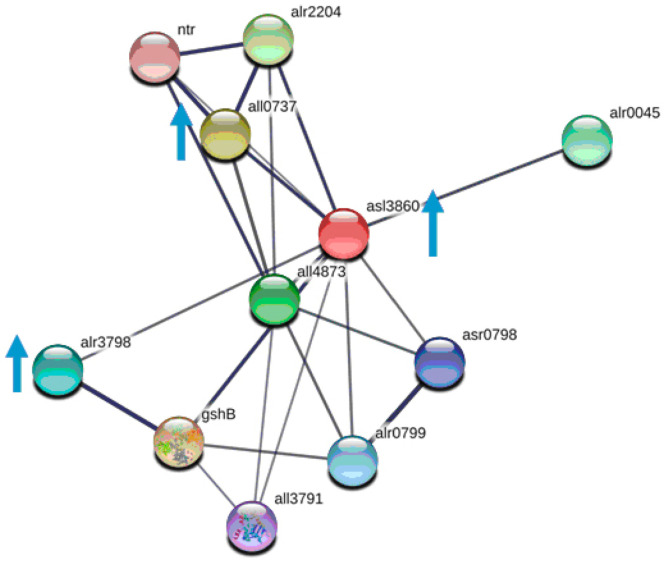
This scheme presents the protein network of Glutaredoxin-3 (asl3860) (red ball) and its protein partners, according to STRING. The protein network is represented with the following 10 protein partners: arl0045 is ferredoxin; asr0798 is a hypothetical protein; alr0799 is monothiol glutaredoxin; all3791 is Ribonuclease D; gshB is Glutathione synthetase (all3859); alr3798 is Glutathione S-transferase; alr2204 and all0737 are Thioredoxin reductases; all4873 is Glutaredoxin-3; and ntr (all4510) is NADP-thioredoxin-reductase.

Several proteins that are part of the central redox machine [83], which contains at least six main protein families, such as thioredoxin reductases (TrxRs), thioredoxins (Trxs), peroxiredoxins (Prxs), glutathione reductases (GRs), glutaredoxins (Grxs), and glutathione peroxidases (Gpxs), were upregulated in *Nostoc* PCC 7120 cells due to BMAA action. One of them, Glutaredoxin-3 (*asl3860*), which is known to have a chaperone function and take part in protein-folding catalysts, was more than fourfold upregulated under BMAA treatment (Table 2). It should be mentioned that glutaredoxins (also known as thioltransferases) are small redox enzymes that use glutathione as a cofactor. This oxidation repair enzyme is also found in human bodies, and is known to participate in many cellular functions, including redox signaling and glucose metabolism regulation. Glutaredoxins are oxidized by substrates and are reduced non-enzymatically by glutathione. Contrary to thioredoxins, which are known to be reduced by thioredoxin reductase, an oxidoreductase that could specifically reduce glutaredoxins is unknown. Glutaredoxins are reduced by the oxidation of glutathione. Glutaredoxin-3 forms interrelations with other enzymes (Figure 3). Two of them (glutathione S-transferase, *alr3798*, and thioredoxin reductase, *all0737*) have also been found as upregulated proteins in our study (Table 2). Mammalian glutaredoxin 3 (Grx3) is critical for maintaining redox homeostasis and essential for the defense mechanism against oxidative stress [84]. A large list of Glutaredoxin-3 associated proteins of *Candida albicans* was identified by using a proteomic approach [85]. These proteins were found to have diverse functions, including iron-sulfur trafficking, iron homeostasis, metabolism redox homeostasis, protein translation, DNA maintenance, and repair. The authors concluded that Glutaredoxin-3 is a global regulator of iron homeostasis and of other iron-dependent cellular processes [85]. Glutaredoxin-3 is significant in transcriptional iron regulation and in intracellular iron distribution. In our study, ABC transporter iron-binding protein (*alr3938*) involved in high-affinity iron ion transport was downregulated in the presence of BMAA (Table 2). This protein is interconnected with other proteins related to iron transport (Supplementary Figure S9). Iron is an essential component of electron transport and is particularly important to photoautotrophs, like cyanobacteria, because 22–23 irons are required for complete functionality of photosynthetic apparatus [86]. Earlier in Section 2.3, we have highlighted the downregulation of four enzymes involved in porphyrin and chlorophyll metabolism that required irons. Moreover, iron is required for nitrogen fixation [87,88]. The nitrogenase protein complex is composed of iron-rich proteins, including NifH (four iron atoms per homodimer) and NifDK (15 iron atoms per homodimer) [89,90]. In addition, besides iron, sulfur is also required for nitrogenase activity [91]. In this study, we found that sulfur metabolism may be impaired under BMAA treatment due to the fact that an enzyme called phosphoadenosine phosphosulfate reductase [EC:1.8.4.8 1.8.4.10] was downregulated (Table 2).

Therefore, the downregulation of nitrogenase molybdenum-iron protein subunits—α (nifD) and β (nifK), as well as the downregulation of iron transport protein and an enzyme involved in sulfur metabolism under BMAA treatment ought to be the cause behind the starvation of cyanobacterial cells and the uprise of corresponding stress. Indeed, in the presence of BMAA the starvation-inducible DNA-binding protein (*all4145*) was found upregulated (Table 2). Moreover, this probable DNA-binding stress protein interacts with several other stress-inducible proteins (Figure S10). According to the data from STRING, among the starvation-inducible DNA-binding protein’s partners, there are dpsA (alr3808), GltS (alr4344), all0457, all0459, all4144, asl4146, all4142, all4143, all0404 and asl4325. Protein dpsA (alr3808) is a nutrient stress-induced DNA-binding protein that is involved in the protection of chromosomal DNA from damage under nutrient-limited and oxidative stress conditions and that binds heme. Protein GltS (alr4344) is a ferredoxin-glutamate synthase that is involved in glutamate biosynthetic process. Proteins all0457 and all0459 are low temperature-induced proteins. Protein all4144 is probably a chaperon. Protein asl4146 is a hypothetical protein with ParB/Sulfiredoxin domains. It should be noted that protein ParB is involved in chromosome partition; it is localized at both poles of a pre-divisional cell and it binds to the DNA origin replication. Sulfiredoxin-1 contributes to the oxidative stress resistance by reducing cysteine-sulfinic acid, which was formed under the exposure to oxidants in peroxiredoxins PRDX1, PRDX2, PRDX3, and PRDX4. Proteins all0404 and asl4325 represent ClpS adapter protein of ATP-dependent Clp protease, which is encoded by two different genes *all0404* and *asl4325*. ClpS adapter protein is involved in the modulation of specific degradation of the ClpAP-mediated ATP-dependent protein.

**Figure 4:**
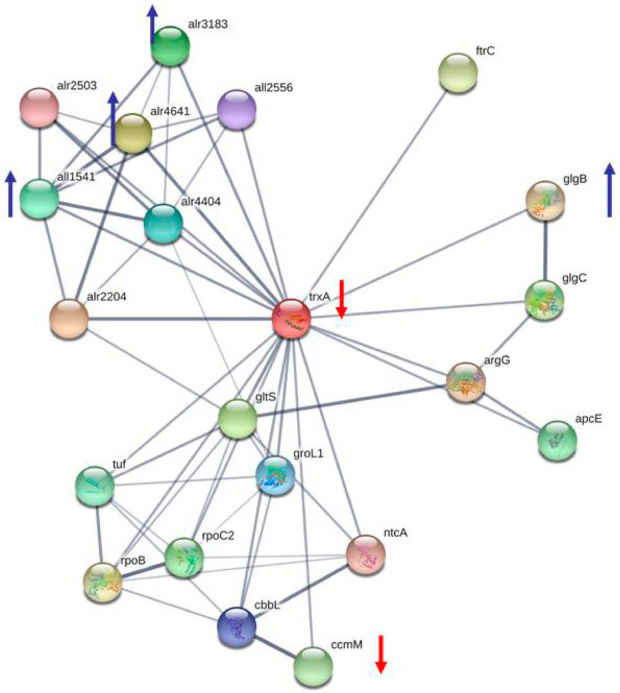
The protein network of thioredoxin TrxA (alr0052) and its protein partners, according to STRING. In this figure protein alr4641 is Peroxiredoxin 2/4 (thioredoxin-dependent peroxiredoxin); alr2204 is Thioredoxin reductase; all1541 is Peroxiredoxin 2 family protein/glutaredoxin; alr3183 is peroxiredoxin Q/ Bacterioferritin comigratory protein; gltS is Ferredoxin-glutamate synthase. In this figure six proteins are pointed out, four of them were upregulated (marked by blue arrows) and two proteins were downregulated (shown by red arrows) under BMAA treatment.

One protein, thioredoxin 1 (trxA, *alr0052*), was found four-fold downregulated under BMAA treatment of *Nostoc* PCC 7120 cells in diazotrophic conditions (Table 2). In the previous proteomic study [24] thioredoxin 1 (*all1866*) was found only in control samples and was not found in BMAA treated cells of *Nostoc* PCC 7120. It was shown that each cyanobacterial genome encodes several thioredoxins (from one to eight thioredoxins), as well as all components that are necessary for the reduction of thioredoxins [92]. Thioredoxins or related thiol-containing proteins catalyze disulfide/dithiol exchange in proteins, which are involved in the assimilation and storage of nutrients, as well as in some central metabolic pathways [92]. Twenty-six trxA-linked proteins have been identified in *Synechocystis* sp. PCC 6803 [93]. Among them were found eighteen cytosolic proteins and eight peripheral membrane proteins. Later seventy-seven thioredoxin target proteins have been identified in *Synechocystis* sp. PCC 6803, and it was suggested that the thioredoxin-mediated redox signaling is equally significant for both oxygenic photosynthetic prokaryotes and oxygenic photosynthetic eukaryotes [94]. Experimental evidence confirmed that thioredoxins have a regulating function in the reductive pentose phosphate cycle in cyanobacteria, which contain the ferredoxin/thioredoxin system, was obtained [95]. Nineteen proteins among the proteins identified in *Nostoc* sp. PCC 73102 were predicted to be thioredoxin targets. It was found that among them were fructose-1,6-bisphosphatase, translation elongation factors, the Rubisco large subunit, chaperones, ATPase, and peroxiredoxins [96]. In our proteomic study, we have found three proteins-partners of TrxA that were upregulated in the presence of BMAA (Table 2). In Figure 4, the protein network of thioredoxin TrxA (alr0052) and its protein partners is presented according to STRING (https://string-db.org). The following proteins were found to be upregulated under BMAA treatment: Peroxiredoxin (*alr4641*); Peroxiredoxin 2 family protein/glutaredoxin (*all1541*) and the peroxiredoxin Q/ Bacterioferritin comigratory protein (*alr3183*) (Table 2).

The detoxification of reactive oxygen species (ROS) is essential for proper cyanobacteria cell functioning. It is important to note one more aspect before discussing the mechanisms of protection against oxidative stress in these photosynthetic organisms. It is known [97] that there is a crosstalk between ROS homeostasis and nitrogen metabolism in cyanobacteria caused by a separate mechanism that is independent of known redox regulators. It was demonstrated that hydrogen peroxide alters the expression of several genes, which are related to nitrogen metabolism, in the wild type of *Synechocystis* sp. PCC 6803 and its mutant that was impaired in the catalase-peroxidase activity and, therefore, was highly sensitive to oxidative stress. It was shown that hydrogen peroxide interferes with the carbon-to-nitrogen ratio status signaling in the cyanobacteria cells by reducing the intracellular concentrations of 2-OG and, consequently, changing the function of the 2-OG-sensitive global nitrogen regulator NtcA [97]. In this work and in the previous proteomic studies [23,24] BMAA induces a strong stress response and changes in the expression of many NtcA regulated proteins (Table 2).

**Figure 5:**
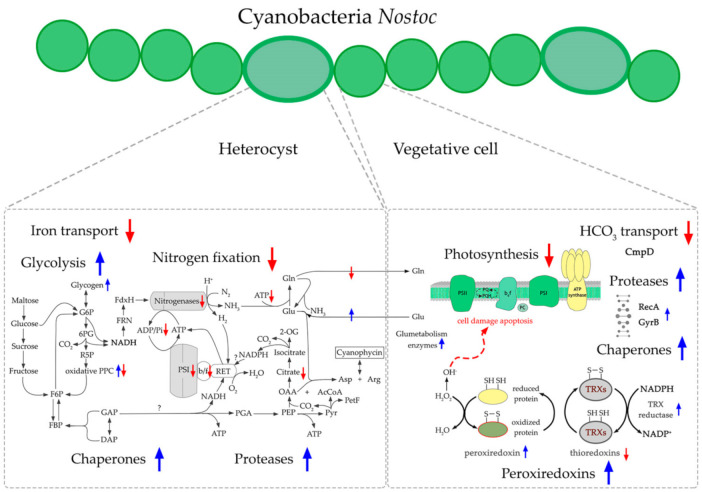
This scheme shows the main effects of BMAA on heterocyst and vegetative cells. Blue arrows indicate upregulated proteins and upregulated processes, while red arrows indicate downregulated protein and downregulated processes. Some components of this scheme were based on schemes from KEGG data base [98].

Summarizing the facts mentioned above, we can state that the main enzymes involved in a redox-control system of cyanobacteria cells were affected by BMAA treatment. Considering the fact that the members of the central redox machine members have cell pleiotropic functions [82], such as detoxification of harmful amounts of reactive oxygen species, they can also act as sensors catching the changes in oxidant concentrations; as well some peroxiredoxins can express chaperone activity. It should be highlighted that BMAA’s impact is significant and has severe consequences for cellular metabolism and DNA functioning consequences. This is supported by the fact that four SOS-responses DNA repair enzymes were upregulated in BMAA treated samples (Table 2) (Figure 5).

The RecA (recombinase A, *all3272*) protein was found 4.5 fold upregulated in the presence of BMAA. This enzyme was upregulated three-fold in nitrogen starving cells [23] and almost fourfold—in nitrogen-replete *Nostoc* PCC 7120 cells under BMAA treatment [24]. Upregulation of two subunits of the DNA gyrase enzyme was found in both proteomic studies (Table 2 and [23]). One more protein, single-stranded DNA-binding protein (*alr0088*), was found upregulated in the current study (Table 2). Among its protein-partners (Supplementary Figure S11) there are proteins, which are involved in DNA repair. One of them is the protein UvrA of the UvrABC system that catalyzes the recognition and processing of DNA lesions. UvrA is an ATPase and a DNA-binding protein. A damage recognition complex, which is composed of 2 UvrA and 2 UvrB subunits, scans DNA for abnormalities. When UvrB has verified the presence of a lesion, the UvrA molecules dissociate. Another protein partner of single-stranded DNA-binding protein (*alr0088*) is the LexA repressor. It represses several genes that are involved in DNA damage response (SOS response), including *recA* and *lexA*. In the presence of single-stranded DNA, RecA interacts with LexA causing an autocatalytic cleavage, which disrupts the DNA-binding part of LexA, leading to derepression of the SOS regulon and eventually to DNA repair. DNA polymerase I is also a partner of the single-stranded DNA-binding protein (*alr0088*). This DNA polymerase exhibits both polymerase activity and 5′-3′ exonuclease activity. The Primosomal protein N’ (pri) is another protein-partner of *alr0088* that is known to recognize and bind the arrested nascent DNA chain at stalled replication forks. It can open the DNA duplex via its helicase activity, promote primosome assembly and boost loading of the major replicative helicase DnaB onto DNA.

Two hypothetical proteins that may be involved in DNA metabolism and DNA recombination (*alr4504* and *alr4505*) (Supplementary Figure S12) were strongly upregulated by BMAA (Table 3). For example, the protein encoded by *alr4505* was found upregulated 20 fold (!).

It can be suggested that oxidative stress, induced by BMAA, may lead to DNA damage and, therefore, may influence the activities of both DNA cell repair and toxin-antitoxin systems.

### 2.9. Hypothetical Proteins

Hypothetical proteins have been identified in *Nostoc* PCC 7120 by using the National Center for Biotechnology Information (NCBI) GenBank Protein Sequence Database. The experimental discovery of hypothetical proteins in the proteome of *Nostoc* PCC 7120 is an essential fact, since it means that these proteins actual exist in the cells. Initially, when these proteins were annotated in the genome, their existence was only theoretically predicted. However, the proteomic method allows researchers to identify them in samples. These proteins have no resemblance to the previously characterized proteins and therefore are called hypothetical. Researchers are often disappointed when they discover hypothetical proteins in the proteome, because the functions of these proteins are unknown. We have used the existing public bioinformatics databases (Section 4.5), with the help of which we try to find the clues to the understanding of their possible functions. The next step that required in order to confirming the functions of hypothetical proteins is to perform mutagenesis, i.e., to inactivate the genes encoding these proteins and to use transcriptome analysis (this is a task for further research studies).

Twenty-six hypothetical proteins have been identified as regulated under BMAA treatment in *Nostoc* PCC 7120 cells during diazotrophic growth (Table 1 and Table 3, Supplementary Table S2). Twenty proteins were upregulated, and six proteins were downregulated. Five hypothetical proteins encoded by *alr4505*, *alr4504*, *asl4547*, *all1411,* and *all3826* were under NtcA regulation (Table 3). Hypothetical proteins encoded by genes *alr4505*, *all1411,* and *asl4547* have been strongly upregulated by BMAA in all three growth conditions [23,24] (Table 3 and Table 4). As we mentioned above, protein *alr4505* in this study was upregulated 20 fold (!) in the presence of BMAA. According to the information available about its protein partners (Supplementary Figure S12), this protein and its partner protein, which is encoded by *alr4504* may be involved in DNA metabolism and DNA recombination and a toxin-antitoxin system. According to the STRING database, asl4547 protein has fellow proteins among its protein partners, such as ABC transporter ATP-binding protein; transmembrane uncharacterized protein; hypothetical protein contained exonuclease VII domain and an AbrB family transcriptional regulator (Supplementary Figure S13). Protein all1411 is involved in functional relations with two uncharacterized proteins, one of which is noted as a membrane protein (Supplementary Figure S14). Protein all3826 is interconnected with N-acetyltransferase domain-containing protein, a protein with Peptidoglycan-bd-like domain, and with membrane proteins and unknown proteins (Supplementary Figure S15). Some useful information about certain hypothetical proteins can be found in ALCOdbCyano database (http://alcodb.jp/cyano/), where co-expressed genes are listed and presented (Supplementary Table S2). Information about co-expressed genes may provide the clues for understanding the possible function of a hypothetical protein. Moreover, we have found that several identified hypothetical proteins were in the same co-expressed gene list with other proteins that also were identified in the present proteomic study (Supplementary Table S2). The application of genetic and transcriptomic approaches may help define these hypothetical proteins’ cell functions.

**Figure 6:**
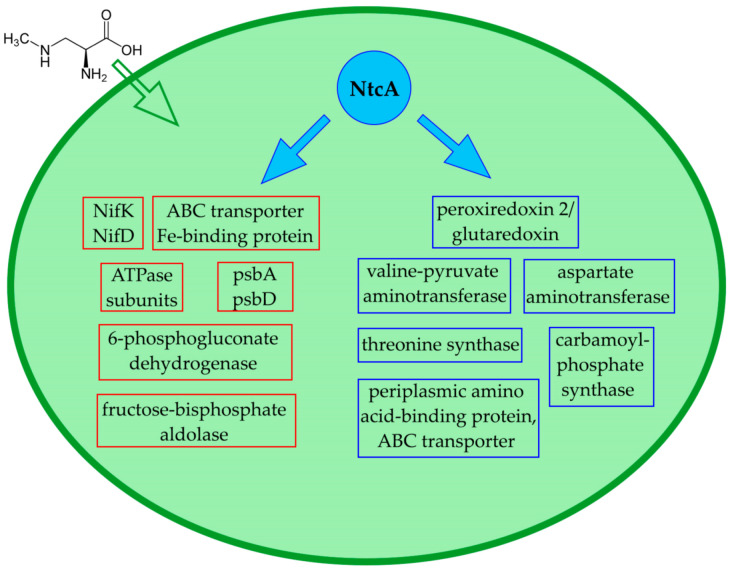
This scheme shows the effects of BMAA on proteins encoded by genes that are under transcription control of the global nitrogen regulator NtcA. Downregulated proteins are highlighted with a red frame and upregulated proteins are highlighted with a blue frame.

**Figure 7:**
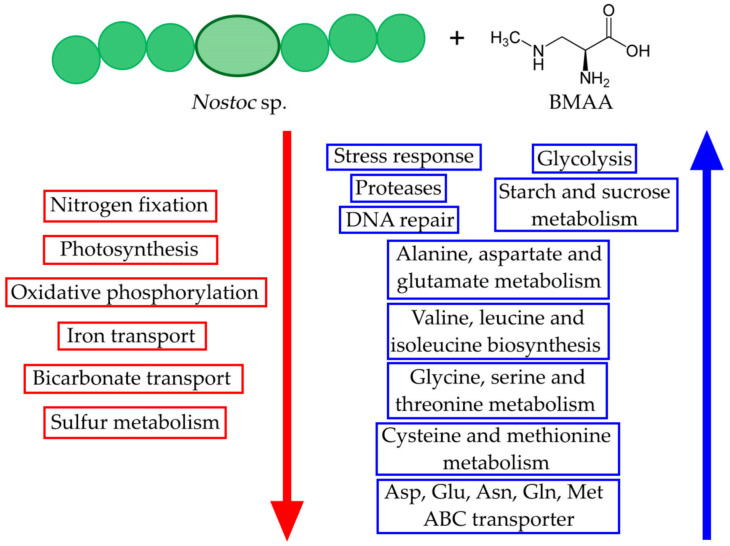
This scheme shows the effects of BMAA on the main metabolic pathways in *Nostoc* PCC 7120 cells under diazotrophic growth conditions. The red arrow shows downregulation, the blue arrow shows upregulation.

The performed proteomic study’s presented results show a significant regulatory effect of β-N-methylamino-L-alanine (BMAA) on cyanobacterium *Nostoc* PCC 7120 proteome under diazotrophic conditions (Figure 6 and Figure 7). The new proteomic data confirms and explains previously published experimental results [17,23] by demonstrating that BMAA disturbs proteins involved in nitrogen fixation and nitrogen metabolism.

Downregulation of the nitrogen-fixation system caused by BMAA leads to N/C dis-balance that induces the decrease of both photosystem proteins. So, besides nitrogen fixation, BMAA downregulates eighteen proteins of both photosystems and light-harvesting complexes, as well as five subunits of ATP synthase and enzyme NAD(P)H-quinone oxidoreductase. As a consequence of these dramatic changes, disbalance in energy and metabolic imbalance originates in intracellular oxidative stress that leads to the activation of several key biomolecules, such as starvation-inducible DNA-binding protein, nine oxidative stress-response enzymes, four proteases, and four SOS-response and DNA repair enzymes, including RecA enzyme. This stress-response detected under diazotrophic conditions in *Nostoc* PCC 7120 cells was more powerful and more evident compared to the stress-responses that were previously observed under BMAA treatment in nitrogen-limited conditions [23] and nitrogen-replete conditions [24] (Figure 7). Therefore, BMAA induces intracellular stress in all growth conditions, but its impact is more harmful in the most nitrogen-poor growth environment.

**Figure 8:**
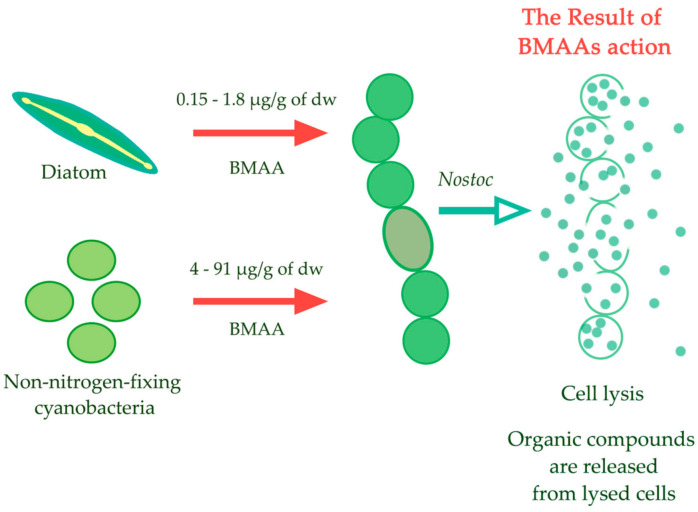
The scheme represents a hypothesis that suggests that BMAA is used as an “allelopathic tool” in the fight for organic nitrogen. Phytoplankton species could use BMAA as a possible allelopathic tool to control cyanobacteria cell populations during periods of strong competition for nitrogen and other nutrient sources in the microalgae community. Cyanobacteria cells undergo lysis in the presence of exogenous BMAA and as a result, dissolved organic compounds, which the algae community needs, are released.

Summarizing the results of the proteomic analysis of the action that BMAA causes on cyanobacteria cells, which are grown under different nitrogen supply conditions (in studies [23,24] and the present study), we can propose that the main primary targets of BMAA action are, apparently, metabolic processes, such as the nitrogen fixation, and different biosynthetic processes, whose regulation involves 2-oxoglutarate, glutamate, regulatory proteins PII and NtcA. Recently it was shown that even in a very small concentration (5 µmol), which do not have a noticeable effect on the growth of unicellular cyanobacteria *Synechococcus* sp. TAU-MAC 0499, BMAA affects the nitrogen assimilation in the treated cyanobacterium [99]. It should be noted that the effect that BMAA causes on nitrogen-fixing cyanobacteria and non-nitrogen-fixing cyanobacteria appears to be different. According to our unpublished observations, growth of the unicellular non-diazotrophic cyanobacteria *Synechococcus elongatus* PCC 7942 and *Synechocystis* sp. PCC 6803 is not affected by BMAA in concentrations of 150–250 µMol. These observations are in agreement with the data recently published in a study [16]. It means that these non-nitrogen-fixing cyanobacteria can metabolize exogenous BMAA that was added to the medium in such high concentrations. Finding out what is the mechanism underlying this observation is another interesting subject for an experimental investigation. We hypothesized before [18,23] and we want to update this hypothesis now that due to the facts that BMAA synthesis is induced by nitrogen starvation of non-nitrogen-fixing unicellular cyanobacteria [13] and this amino acid in micromolar amounts (20–50 µmol) strongly affects the availability of nitrogen-fixing strains [15,16,17,18,23,24], BMAA could be used by phytoplankton (diatom, cyanobacteria, and dinoflagellates) as a possible allelopathic tool to control cyanobacteria cell populations during the periods of strong competition for nitrogen and other resources in the microalgae community. We can assume that diazotrophic cyanobacteria cells undergo lysis under exogenous BMAA action, and dissolved organic compounds that are necessary for the algae community are released (Figure 8).

## 3. Conclusions

This work has demonstrated for the first time that β-N-methylamino-L-alanine (BMAA) causes a severe stressful effect on cyanobacterium *Nostoc* sp. PCC 7120 proteome under diazotrophic conditions. Moreover, cyanotoxin BMAA strongly downregulates the α (NifD) and β (NifK) subunits of molybdenum-iron protein (component I) in nitrogenase. The corresponding genes (*nifD* and *nifK*) are under transcriptional control of the global transcriptional regulator NtcA in cyanobacteria. Furthermore, it was shown that these two genes are not the only ones under the control of the NtcA that are being affected by BMAA. BMAA impacts a total of twenty-two proteins that are under the transcriptional control of NtcA. Among them are nitrogenase structural proteins, ABC transporters, key components of photosystem II and ATPase, amino acid metabolism enzymes, carbohydrate metabolism enzymes, and peroxiredoxins. Besides that, BMAA induces a high upregulation of stress-activated proteins, such as oxidative stress enzymes (e.g., peroxiredoxins, glutaredoxin) and SOS response DNA repair enzymes (e.g., RecA, GyrB). 

Thus, the obtained proteomic data shows a broad picture of regulatory changes that are induced by BMAA in the cyanobacteria metabolic networks. This information is essential for further fundamental investigations of this toxic amino acid’s regulatory roles in cyanobacteria cells. Moreover, the presented data provides new leads into the ecological impact of BMAA on microalgal communities that can be used in future investigations.

## 4. Materials and Methods

### 4.1. Cyanobacterial Strain and Cultivation Conditions

Filamentous nitrogen-fixing cyanobacterium *Nostoc* sp. PCC 7120 was grown in 100 mL Erlenmeyer flasks containing 25 mL of BG11_0_ medium [100] for 3 days on a shaker with continuous shaking at the rate of 63 rpm and at a light intensity of 18 µmol photons m^−2^s^−1^ and at 25 °C. Afterwards cells were collected and washed 3 times with a nitrogen-free medium (BG11_0_). After that, cyanobacterium was grown in BG11_0_ medium for 48 h in two experimental versions, in which: (1) the control samples were grown without the addition of aqueous BMAA solution and (2) the treated samples were grown with the addition of a water solution of β-N-methylamino-L-alanine (L-BMAA) (Cat no. B-107, Sigma-Aldrich, Saint Louis, MO, USA) in a final concentration of 20 µM, as it was performed earlier in [23,24]. Later, cells from both experimental sets were collected by centrifugation at 5000 rpm for 10 min at 4 °C and were frozen at −80 °C until they were used for the proteomics analysis. The experiment was performed in 3 independent biological replicates.

The time period during which cells were treated with BMAA (48 h) was selected according to [23,24].

### 4.2. Trypsin Digestion in Solution

Lysozyme (0.3 mg/mL) (Sigma, Saint Lois, MO, USA) was added to each cellular pellet and gently mixed. Then the mixture was incubated for 60 min at 4 °C. Afterwards the mixture was resuspended in 100 µL of 100 mM tris-HCl buffer, pH 8.0, with the addition of Protease inhibitor Mix (GE Healthcare, Chicago, IL, USA), 0.1% sodium deoxycholate (DCNa) (Sigma, Saint Lois, MO, USA) and 2.5 mM EDTA (Sigma, Saint Lois, MO, USA). In order to perform full cell lysis six sonication cycles of 30 s (Cell Disruptor, Branson, Branson Ultrasonics Corp., Danbury, CT, USA) were performed and followed by a short 5 min incubation period at 4 °C. After that, dry urea and DCNa were added to the sample to obtain the final concentrations of 8 M and 1%, respectively. After an incubation period of 20 min, the sample was centrifuged at 14,000 rpm for 10 min at 4 °C to remove intact cells. The supernatant was collected, and the protein concentration was estimated by using Bradford Protein Assay Kit (BioRad, Hercules, CA, USA). To reduce the cysteine bonds of proteins in the supernatant 5 mM Tris (2-carboxyethyl) phosphine hydrochloride (TCEP) (Sigma, Saint Lois, MO, USA) was added, the mixture was incubated for 60 min at 37 °C and, subsequently alkylated with 30 mM iodoacetamide (BioRad, Hercules, CA, USA) during 30 min at a room temperature in the dark. Then the step, in which TCEP was added, was repeated. Afterwards the sample was diluted 6-fold with 50 mM Tris-HCl, pH 8.0, with the 0.01% DCNa. Trypsin (Trypsin Gold, Mass Spectrometry Grade, Promega, Madison, WI, USA) was added in 1/50 *w/w* trypsin/protein ratio and incubated at 37 °C overnight. Trypsinolysis was stopped and the acid-labile DCNa was degraded by the addition of trifluoroacetic acid (TFA) (Sigma, Saint Lois, MO, USA) to a final concentration of 0.5% *v/v* (the pH must be less than 2.0) and agitation for 45 min at 37 °C. Afterwards the sample was centrifugated at 14,000× *g* for 10 min to remove the DCNa. The peptide extract was desalted by using a Discovery DSC-18 Tube (Supelco, Merck KGaA, Darmstadt, Germany) according to the manufacturer’s protocol. Peptides were eluted with 1 mL 75% acetonitrile (Sigma, Saint Lois, MO, USA) and 0.1% TFA and dried in a benchtop vacuum concentrator SpeedVac (Labconco, Kansas City, MO, USA) and resuspended in 3% acetonitrile with 0.1% TFA to a final concentration of 5 μg/μL.

### 4.3. LC-MS/MS Analysis

The analysis was performed on a Triple TOF 5600+ mass spectrometer with a NanoSpray III ion source (AB Sciex, Framingham, MA, USA) coupled with a NanoLC Ultra 2D+ nano-HPLC system (Eksigent, now part of Sciex, Framingham, MA, USA) as we have described in [101]. The HPLC system was set in a trap-elute mode. The buffer A and the sample loading buffer consist of a mixture of 98.9% water, 1% methanol, 0.1% formic acid (*v/v*). Buffer B included 99.9% acetonitrile and 0.1% formic acid (*v/v*). Samples were loaded on a Chrom XP C18 trap column (3.6 μm, 120 Ǻ, 350 μm × 0.5 mm; Eksigent) at a flow rate of 3 μL/min for 10 min and eluted through a 3C18-CL-120 separation column (3 μm, 120 Ǻ, 75 μm × 150 mm; Eksigent) at a flow rate of 300 nL/min. The gradient was performed from 5% to 40% buffer B during 90 min followed by a 10 min period at 95% buffer B and 20 min period of re-equilibration with 5% buffer B. To wash the system and in order to prevent carryover, two blank 45-min runs, which consisted of several waves (5% B, 95%, 95%, 5%) that persisted from 5 to 8 min, were performed between the different samples.

The information-dependent mass-spectrometer experiment included one survey MS1 scan that was followed by 50 dependent MS2 scans. MS1 acquisition parameters were set as follows: the mass range for MS2 analysis was 300–1250 *m*/*z*, and the signal accumulation time was 250 ms. Ions for MS2 analysis were selected on the basis of intensity with a threshold of 200 counts per second and a charge state from 2 to 5. MS2 acquisition parameters were the following: the resolution of the quadrupole was set to UNIT (0.7 Da), the mass measurement range was 200–1800 *m*/*z*, and the signal accumulation time was 50 ms for each parent ion. Collision-activated dissociation was performed with nitrogen gas, and the collision energy ranged from 25 to 55 V within the signal accumulation time of 50 ms. Analyzed parent-ions were sent to the dynamic exclusion list for 15 s in order to get MS2 spectra at the chromatographic peak apex.

β-Galactosidase tryptic solution (20 fmol) was run with a 15-min gradient (5% to 25% buffer B) between every two samples and between sample sets to calibrate the mass spectrometer and in order to control the overall system performance, stability, and reproducibility.

### 4.4. Protein Identification by Using LC-MS/MS Data Analysis

In order to fulfill protein identification and semi-quantitative spectral counting, all LC-MS/MS data were searched against the National Center for Biotechnology Information (NCBI) GenBank Protein Sequence Database for *Nostoc* sp. PCC 7120. Identification of proteins was performed with ProteinPilot (version 4.5, Sciex, Framingham, MA, USA, 2012) in an identification mode with the following parameters: Cys alkylation performed with iodoacetamide; trypsin digestion; TripleTOF 5600 instrument; false discovery rate (FDR) analysis; and a thorough ID search with a protein detection threshold of 95.0%. Protein identification was considered significant if the estimated local false discovery rate was 1% or lower, and at least 2 different peptides in the protein were identified with a confidence score above 95%. Spectral counting was performed with in-house script under emPAI protocol [102] with only tryptic peptides with local FDR ≤ 1%.

Quantitative analysis was performed with MaxQuant (version 1.4.3.14, Max Planck Institute of Biochemistry, Munich, Germany, 2014) [103] against the same database. The settings used were as follows: a standard label-free analysis; fixed cysteine carbamidomethylation (which is permitted to be used in quantitative analysis); no variable modifications; default settings for AB Sciex Q-TOF instrument during MS and MS/MS spectra processing; tryptic digest with KP/RP cleavage were to be prohibited with 0 missed sites allowed; label-free quantification was set with minimum 2 label-free quantification (LFQ) ratios; normalization was performed and missing peaks were re-quantified; the minimum peptide length was 7, the maximum peptide mass was set as 4600 Da, only unique peptides were to be used for quantification. The PSM and protein FDR threshold were set at 5%, and at least 1 unique peptide was required for the protein group. The statistical significance of the observed differences was assessed in each case with Welch’s 2-sided t-test that was adjusted with Benjamini-Yekutieli adjustment for multiple comparisons, which was used to compare the obtained *p*-value with *p*-value thresholds of 0.05 and 0.1.

### 4.5. Pathway Analysis Based on LC-MS/MS Data

The significantly altered proteins obtained from LC-MS/MS data analysis were subjected to analysis The proteins that were noticed to be significantly altered in the of presents BMAA according to accomplished LC-MS/MS data analysis were, therefore, subjected to analysis/analyzed/subjected to be analyzed//by using the UniProt Knowledgebase (https://web.expasy.org/docs/userman.html#what_is_sprot) and the Kyoto Encyclopedia of Genes and Genomes (KEGG) pathways database (https://www.genome.jp/kegg/pathway.html).

Protein-Protein interactions were analyzed by the Protein-Protein Interaction Networks Functional Enrichment Analysis; https://stringdb.org/cgi/download.pl?sessionId=LdNVdFoNwm9Q). Gene co-expression data assembled for *Nostoc* (*Anabaena*) sp. PCC 7120 were obtained from ALCOdbCyano (http://alcodb.jp/cyano/). The co-expression data in this database were calculated by using 116 microarray data items downloaded from the KEGG EXPRESSION Database (https://www.genome.jp/kegg/expression/). Sequence information and gene annotations were retrieved from CyanoBase (http://genome.microbedb.jp/mnt.html).

NtcA-regulated genes were found with CollecTF database (a database of transcription factor binding sites (TFBS) in the Bacteria domain) (http://www.collectf.org/browse/home/) [25].

## Figures and Tables

**Table 1:** The effect of beta-N-methylamino-L-alanine (BMAA) on the protein profile of *Nostoc* PCC 7120 during its growth in diazotrophic conditions. The number of upregulated and downregulated proteins is quantified according to the label-free quantification ratio (sample treated with BMAA)/(control sample).

№	Pathway	Number of Proteins Affected by BMAA	Total Amount	
			Up-Regulated	Down-Regulated
1	Heterocyst formation and functionality	3	1	2
2	Photosynthesis	19	1	18
3	Oxidative phosphorylation	6	0	6
4	CO_2_-concentrating mechanism	2	0	2
5	Carbohydrate metabolism	14	8	6
6	Transporters	3	1	2
7	Sulfur metabolism	1	0	1
8	Secondary metabolites	4	1	3
9	Proteases	5	4	1
10	Chaperones	3	2	1
11	Stress response	11	9	2
12	SOS-response and DNA repair	4	4	0
13	Transcription	2	2	0
14	Translation	8	4	4
15	Amino acid synthesis and metabolism	11	10	1
16	Purine and pyrimidine metabolism	1	1	0
17	Hypothetical proteins	26	20	6
	Total	123	68	55

**Table 2:** The effect of BMAA on the protein profile of *Nostoc* PCC 7120 during diazotrophic growth (fold changes between treated samples and control samples are shown, (*p* < 0.1)). *p*-value: * < 0.1, ** < 0.05, *** < 0.01. ^#^ Genes are under NtcA transcriptional regulation.

№	Protein	Gene	Function	Up Shifted	Down Shifted	*p*-Value
Nitrogen fixation and heterocyst formation (3 proteins)						
1	NifK	*all1440 ^#^*	nifK|nitrogenase molybdenum-iron protein subunit β		0.199	0.0021 ***
2	nifD	*all1454 ^#^*	molybdenum-iron protein subunit α in nitrogenase		0.59	0.0131 **
3	Apb2	*all1939*	transcription regulation of *hepA* and *hepC* genes	1.49		0.0286 **
ABC-transporters and transporters (3 proteins)						
4	Alr3938	*alr3938 ^#^*	ABC transporter iron binding protein (high-affinity iron ion transport)		0.67	0.0771 *
5	YidC	*alr3415*	inner membrane protein translocase component YidC		0.81	0.0105 **
6	alr4164	*alr4164 ^#^*	periplasmic amino acid-binding protein of amino acid ABC transporter	1.47		0.0469 **
CO_2_-concentrating mechanism and bicarbonate transport (2 proteins)						
7	ccmM	*all0865*	CcmM, carbon dioxide concentrating mechanism protein		0.70	0.0126 **
8	cmpD	*alr2880*	CmpD, bicarbonate transport ATP-binding protein		Found only in control sample	0.0349 **
Proteases (5 proteins)						
9	alr2758	*alr2758*	serine proteinase		0.67	0.0259 **
10	carboxyl-terminal processing protease [EC:3.4.21.102]	*all2500*	carboxyl-terminal protease (serine endopeptidase)	1.43		0.0279 **
11	ClpP (subunit 1) [EC:3.4.21.92]	*alr1238*	ATP-dependent Clp P protease proteolytic subunit, ATP-dependent Clp protease proteolytic subunit 1	1.59		0.0338 **
12	ClpP (subunit 2) [EC:3.4.21.92]	*alr3683*	ATP-dependent Clp protease proteolytic subunit, ATP-dependent Clp protease proteolytic subunit 2	1.85		0.0167 **
13	ATP-dependent Clp protease, protease subunit [EC:3.4.21.92]	*all4358*	ATP-dependent Clp protease-like protein	1.69		0.0287 **
Photosynthesis (19 proteins)						
14	photosystem I reaction center subunit IV	*psaE* *asr4319*	photosystem I		0.65	0.0023 ***
15	photosystem I reaction center protein subunit XI	*psaL* *all0107*	photosystem I		0.69	0.0372 **
16	psbA1	*alr4866*; *alr4592*; *alr3727*; *^#^all3572*; *alr3742*	photosystem II protein D1		0.56	0.0065 ***
17	psbB	*all0138*	photosystem II CP47 protein		0.75	0.0034 ***
18	psbD	*alr4548 ^#^ alr4290*	photosystem II protein D2		0.62	0.0563 *
19	psbO	*all3854*	manganese-stabilizing protein		0.81	0.0222 **
20	cpcB	*alr0528*	phycocyanin β chain		0.81	0.0359 **
21	cpcG1	*alr0534*	phycobilisome rod-core linker protein		0.81	0.0671 *
22	cpcG2	*alr0535*	phycobilisome rod-core linker protein		0.61	0.0330 **
23	cpcG4	*alr0537*	phycobilisome rod-core linker protein		0.69	0.0234 **
24	pecB	*alr0523*	phycoerythrocyanin β chain		0.83	0.0873 *
25	hemC hydroxymethylbilane synthase [EC:2.5.1.61]	*alr1878*	Porphyrin and chlorophyll metabolism		0.47	0.0089 ***
26	hemH protoporphyrin/coproporphyrin ferrochelatase [EC:4.99.1.1 4.99.1.9]	*alr3751*	Porphyrin and chlorophyll metabolism		0.53	0.0075 ***
27	protochlorophyllide reductase [EC:1.3.1.33]	*all1743*	Porphyrin and chlorophyll metabolism		0.61	0.0161 **
28	magnesium-protoporphyrin IX monomethyl ester (oxidative) cyclase [EC:1.14.13.81]	*alr3300*	Porphyrin and chlorophyll metabolism		0.52	0.0534 *
29	petH	*all4121*	Ferredoxin-NADP(+) reductase		0.72	0.0187 **
30	petB	*alr3421*	cytochrome b6		0.625	0.0111 **
31	petC	*all2453*	cytochrome b6-f complex iron-sulfur subunit		0.43	0.0844 *
32	cytA	*alr4251*	cytochrome c6	1.69		0.0202 **
Oxidative phosphorylation (6 proteins)						
33	ndhH	*alr3355*	NAD(P)H-quinone oxidoreductase subunit H		0.60	0.0358 **
34	F-type H+-transporting ATPase subunit a	*all0010 ^#^*	ATP synthase F0F1 subunit A		0.65	0.0232 **
35	atpC	*all0004 ^#^*	F-type H+-transporting ATPase subunit gamma		0.63	0.0075 ***
36	atpD	*all0006 ^#^*	ATP synthase F0F1 subunit delta		0.70	0.0090 ***
37	atpF	*all0007 ^#^*	ATP synthase F0F1 subunit B		0.61	0.0879 *
38	F-type H+/Na+-transporting ATPase subunit β [EC:7.1.2.2 7.2.2.1]	*all5039*	ATP synthase F0F1 subunit β		0.76	0.0166 **
Amino acids biosynthesis and metabolism (11 proteins)						
39	urease subunit α [EC:3.5.1.5]	*alr3670*	Arginine biosynthesis Purine metabolism		0.63	0.0059 ***
40	acetolactate synthase I/II/III large subunit [EC:2.2.1.6]	*all3555*	Valine, leucine and isoleucine biosynthesis	1.43		0.0211 **
41	valine-pyruvate aminotransferase [EC:2.6.1.66]	*alr2811 ^#^*	Valine, leucine and isoleucine biosynthesis	1.49		0.0644 *
42	glyA glycine hydroxymethyltransferase [EC:2.1.2.1]	*alr4806*	Glycine, serine and threonine metabolism	1.69		0.0288 **
43	carbamoyl-phosphate synthase large subunit [EC:6.3.5.5]	*alr3809 ^#^*	Alanine, aspartate and glutamate metabolism Pyrimidine metabolism	1.28		0.0496 **
44	adenylosuccinate synthase [EC:6.3.4.4]	*alr4784*	Alanine, aspartate and glutamate metabolism Purine metabolism	1.64		0.0763 *
45	succinate-semialdehyde dehydrogenase/glutarate-semialdehyde dehydrogenase EC:1.2.1.16 1.2.1.79 1.2.1.20	*all3556*	Alanine, aspartate and glutamate metabolism Lysine degradation Tyrosine metabolism	1.67		0.0085 ***
46	nodM glutamine-fructose-6-phosphate transaminase (isomerizing) [EC:2.6.1.16]	*alr3464*	Alanine, aspartate and glutamate metabolism	1.85		0.0013 ***
47	aspartate aminotransferase [EC:2.6.1.1] 2-oxoglutarate-glutamate aminotransferase L-aspartate + 2-oxoglutarate = oxaloacetate + L-glutamate	*alr4853 ^#^*	Alanine, aspartate and glutamate metabolism Arginine biosynthesis Cysteine and methionine metabolism Arginine and proline metabolism Tyrosine metabolism Phenylalanine metabolism Phenylalanine, tyrosine and tryptophan biosynthesis	1.61		0.0016 ***
48	S-adenosylmethionine synthetase [EC:2.5.1.6]	*alr4124*	Cysteine and methionine metabolism	1.54		0.0064 ***
49	threonine synthase [EC:4.2.3.1]	*alr3293 ^#^*	Glycine, serine and threonine metabolism Vitamin B6 metabolism	1.56		0.0599 *
Chaperones (3 proteins)						
50	DnaJ	*alr2447*	molecular chaperone DnaJ	1.35		0.0856 *
51	DnaK	*alr1742*	molecular chaperone DnaK	1.49		0.0176 **
52	GroEL	*alr1896*	molecular chaperone GroEL		0.79	0.0402 **
Stress response (11 proteins)						
53	glutathione S-transferase	*alr3798*	glutathione S-transferase	1.92		0.0014 ***
54	Glutaredoxin-3	*asl3860*	glutaredoxin	4.35		0.0032 ***
55	gor	*all4968*	glutathione reductase	1.30		0.0118 **
56	peroxiredoxin 2 family protein/glutaredoxin	*all1541 ^#^*	peroxiredoxin 2 family protein/glutaredoxin	1.41		0.0918 *
57	peroxiredoxin Q/BCP [EC:1.11.1.15]	*alr3183*	Acting on a peroxide as acceptor	1.69		0.0011 ***
58	peroxiredoxin	*alr4641*	peroxiredoxin	2.27		0.0001 ***
59	thioredoxin reductase	*all0737*	thioredoxin reductase	2.17		0.0241 **
60	peptidylprolyl isomerase [EC:5.2.1.8]	*alr0577*	FKBP-type peptidyl-prolyl cis-trans isomerase	1.52		0.0596 *
61	starvation-inducible DNA-binding protein	*all4145*	probable DNA-binding stress protein	1.45		0.0015 ***
62	trxA|thioredoxin	*alr0052*	trxA|thioredoxin		0.23	0.0205 **
63	FMN-dependent NADH-azoreductase [EC:1.7.1.17]	*all2105*	The biotransformation and/or detoxification of Nitro aromatic compounds can be possible by microbial azoreductase enzyme. Azoreductase enzyme has an ability to reduce the toxic nitro group to the corresponding amino group.		0.70	0.0508 *
SOS-response and DNA repair (4 proteins)						
64	recA	*all3272*	recA|recombinase A	4.55		0.0002 ***
65	DNA gyrase subunit A	*all0860*	DNA gyrase subunit A	1.69		0.0096 ***
66	gyrB|DNA gyrase subunit B	*all5265*	gyrB|DNA gyrase subunit B	2.04		0.0001 ***
67	single-stranded DNA-binding protein	*alr0088*	single-stranded DNA-binding protein	2.04		0.0968 *
Transcription (2 proteins)						
68	antitermination protein NusA	*alr3829*	transcription termination	1.85		0.0022 ***
69	DNA-directed RNA polymerase subunit α [EC:2.7.7.6]	*all4191*	rpoA; RNA polymerase α subunit	1.49		0.0486 **
Translation (8 proteins)						
70	small subunit ribosomal protein S3	*all4209*	rps3; 30S ribosomal protein S3	2.56		0.0017 ***
71	small subunit ribosomal protein S7	*all4339*	30S ribosomal protein S7		0.63	0.0109 **
72	large subunit ribosomal protein L19	*alr5297*	rpl19; 50S ribosomal protein L19		0.37	0.0097 ***
73	rbpD	*asl4022*	RNA-binding protein		0.28	0.0097 ***
74	fus; translation elongation factor EF-G	*all4338*	elongation factor G	1.19		0.0830 *
75	glyS, glycyl-tRNA synthetase β chain [EC:6.1.1.14]	*alr4111*	glycyl-tRNA synthetase β chain	1.35		0.0063 ***
76	aspS, aspartyl-tRNA synthetase [EC:6.1.1.12]	*all2436*	aspartate-tRNA ligase	1.85		0.0342 **
77	phenylalanyl-tRNA synthetase β chain [EC:6.1.1.20]	*alr4958*	phenylalanyl-tRNA synthetase		0.77	0.0431 **
Purine and Pyrimidine metabolism (1 protein)						
78	IMP dehydrogenase [EC:1.1.1.205]	*alr0051*	Purine metabolism	1.45		0.0421 **
Carbohydrate metabolism, Glycolysis and gluconeogenesis, Citrate cycle, Pentose phosphate pathway, Starch and sucrose metabolism (14 proteins)						
79	6-phosphogluconate dehydrogenase [EC:1.1.1.44 1.1.1.343]	*alr5275 ^#^*	Pentose phosphate pathway Glutathione metabolism		0.67	0.0001 ***
80	transketolase [EC:2.2.1.1]	*alr3344*	Pentose phosphate pathway Carbon fixation		0.68	0.0039 ***
81	aconitate hydratase 2/2-methylisocitrate dehydratase [EC:4.2.1.3 4.2.1.99]	*all1267*	Citrate cycle, first carbon oxidation, oxaloacetate => 2-oxoglutarate Glyoxylate and dicarboxylate metabolism		0.65	0.0478 **
82	phosphoglycerate kinase [EC:2.7.2.3]	*all4131*	Glycolysis/Gluconeogenesis Carbon fixation in photosynthetic organisms		0.67	0.0015 ***
83	fructose-bisphosphate aldolase, class II [EC:4.1.2.13]	*all4563^#^*	Glycolysis/Gluconeogenesis Pentose phosphate pathway		0.45	0.0073 ***
84	fructose-1,6-bisphosphatase II/sedoheptulose-1,7-bisphosphatase [EC:3.1.3.11 3.1.3.37]	*alr1041*	Glycolysis/Gluconeogenesis Pentose phosphate pathway		0.55	0.0421 **
85	pyruvate dehydrogenase E1 component beta subunit [EC:1.2.4.1]	*all0122*	Glycolysis/Gluconeogenesis Citrate cycle Pyruvate metabolism	1.19		0.0442 **
86	glucose-6-phosphate isomerase [EC:5.3.1.9]	*alr1050*	Glycolysis/Gluconeogenesis Pentose phosphate pathway Starch and sucrose metabolism	1.49		0.0085 ***
87	Phosphoglucomutase / phosphomannomutase	*all5089*	Glycogenolysis and glycogenesis	1.45		0.0561 *
88	fructose-1,6-bisphosphatase I [EC:3.1.3.11]	*all4021*	Glycolysis/Gluconeogenesis Pentose phosphate pathway Fructose and mannose metabolism	Present only in BMAA treated samples		0.0048 ***
89	glycogen phosphorylase [EC:2.4.1.1]	*all1272*	Starch and sucrose metabolism	2.22		0.0862 *
90	glgB 1,4-alpha-glucan branching enzyme [EC:2.4.1.18]	*all0713*	Starch and sucrose metabolism	1.67		0.0096 ***
91	UDP-glucose 6-dehydrogenase [EC:1.1.1.22]	*alr0658*	Pentose and glucuronate interconversions Ascorbate and aldarate metabolism	1.43		0.0831 *
92	rfbB UDP-glucuronate decarboxylase [EC:4.1.1.35]	*alr0657*	Amino sugar and nucleotide sugar metabolism, Starch and sucrose metabolism	2.5		0.0844 *
Sulfur metabolism (1 protein)						
93	phosphoadenosine phosphosulfate reductase [EC:1.8.4.8 1.8.4.10]	*all4464*	Sulfur metabolism		0.56	0.0071 ***
Secondary metabolites (4 proteins)						
94	(E)-4-hydroxy-3-methylbut-2-enyl-diphosphate synthase [EC:1.17.7.1 1.17.7.3]	*all2501*	Terpenoid backbone biosynthesis		0.53	0.0003 ***
95	15-cis-phytoene desaturase [EC:1.3.5.5]	*alr1832*	Carotenoid biosynthesis		0.76	0.0102 **
96	carboxymethylenebutenolidase [EC:3.1.1.45]	*alr1077*	Dienelactone hydrolase		0.63	0.0479 **
97	NADH dehydrogenase demethylphylloquinone reductase [EC:1.6.5.12]	*alr4094*	Ubiquinone and other terpenoid-quinone biosynthesis	3.33		0.0073 ***

**Table 3:** The effect of BMAA on the protein profile of hypothetical proteins in *Nostoc* PCC 7120 during its growth in diazotrophic conditions (fold changes between treated samples and control samples are shown, (*p* < 0.1)), *p*-value: * < 0.1, ** < 0.05, *** < 0.01. The possible function has been deducted from the BLAST search similarity or the presence of a specific domain (https://www.uniprot.org/). *^#^* Genes are under NtcA transcriptional regulation.

№	Gene	Possible Function	Up Shifted	Down Shifted	*p*-Value
Hypothetical proteins (29 proteins)					
1	*alr4642*	putative thiol-specific antioxidant protein	BTS		0.0628 *
2	*alr7504*	ubiquitin-like modifier activating enzyme activity	BTS		0.0111 **
3	*all4387*	Membrane protease subunit, stomatin/prohibitin	BTS		0.0136 **
4	*alr4505 ^#^*	May be involved in DNA metabolism and recombination	20		0.0014 ***
5	*alr4504 ^#^*	May be involved in DNA metabolism and recombination.	4.35		0.0309 **
6	*all1411 ^#^*	Unknown	5.26		0.0335 **
7	*alr0740*	stomatin-like protein (uncharacterized)	3.13		0.0011 ***
8	*alr7502*	Uncharacterized protein with ubiquitin-like domains	3.03		0.0162 **
9	*all0646*	Thylakoid formation protein Thf1-like protein	2.56		0.0332 **
10	*all2705*	Rho termination factor	2.17		0.0003 ***
11	*all3984*	Conjugal transfer protein TrbI	2.08		0.0041 ***
12	*all0459*	Uncharacterized low temperature-induced protein	2.04		0.0248 **
13	*alr4995*	Saccharop_dh_N domain-containing protein	2.00		0.0014 ***
14	*alr1143*	Uncharacterized protein	1.96		0.0239 **
15	*asl4547 ^#^*	Unknown	1.92		0.00005 ***
16	*all5026*	Short-chain dehydrogenases/ reductases (SDR)	1.67		0.0831 *
17	*alr2055*	unknown	1.54		0.0889 *
18	*alr0114*	Tic22-like family protein involved in the preprotein translocation pore in chloroplasts.	1.49		0.1004 *
19	*all5218*	PmbA; putative modulator of DNA gyrase	1.49		0.0169 **
20	*all3797*	Uncharacterized surface protein containing fasciclin (FAS1) repeats	1.43		0.0232 **
21	*all4296*	AAA domain-containing protein belongs to diverse group of enzymes that are able to induce structural changes in a wide range of substrate proteins and protein complexes		Control	0.0343 **
22	*all1351*	Contains region “OxoGdeHyase_C” (2-oxoglutarate dehydrogenase C-terminal)		0.46	0.0534 *
23	*all7598*	Unknown		0.53	0.0587 *
24	*all3941*	Unknown		0.64	0.0783 *
25	*alr1850*	Phosphoketolase region		0.65	0.0280 **
26	*all3826 ^#^*	Peptidoglycan-binding (PGRP) domain of peptidoglycan hydrolases [Cell wall/membrane/envelope biogenesis		0.67	0.0505 *

«BTS» (**B**MAA **T**reated **S**ample) marks the proteins that were found only in BMAA treated samples; «Control» stands for the proteins that were found only in the control sample.

**Table 4:** The impact of BMAA on proteins that are under NtcA transcriptional control in *Nostoc* PCC 7120. The referred proteins have been identified in different growth conditions.

Pathways and Cell Processes	Nitrogen Starvation (Previous Study [23])		Nitrogen Replete Growth (Previous Study [24])		Diazotrophic Growth (Present Study)	
	Upshifted	Downshifted	Upshifted	Downshifted	Upshifted	Downshifted
Heterocyst formation and functionality		*all1454 nifD*				*all1440 nifK* *all1454 nifD*
Nitrogen metabolism	*alr0608* nrtA	all2319 PII	*all2319* PII			
CO_2_ fixation		*alr1524* RbcL	*alr1524* RbcL *alr1533* RuBisCO Activase	*alr1526* RbcS		
CO_2_ concentrating mechanism		CcmM	CcmK	CmpA		CmpD
Carbon metabolism		*alr5275* 6-phosphogluconate dehydrogenase Pentose phosphate pathway, Glutathione metabolism *alr4566* NADH-dependent butanol dehydrogenase				*alr5275* 6-phosphogluconate dehydrogenase [EC:1.1.1.44 1.1.1.343] *all4563* fructose-bisphosphate aldolase, class II [EC:4.1.2.13]
Photosynthesis		*alr4380* EC:4.2.1.24 delta-aminolevulinic acid dehydratase Porphyrin and chlorophyll metabolism				*alr3727*, *alr3742* psbA photosystem II protein D1 alr4548 psbD photosystem II protein D2
Oxidative phospho- rylation				*all3570* inorganic pyrophosphatase [EC:3.6.1.1]		*all0010* *all0004* *all0006* *all0007* ATP synthase F0F1 subunits
Amino acids metabolism			*all2521* cysteine synthase [EC:2.5.1.47]	*alr2811* valine-pyruvate aminotransferase [EC:2.6.1.66] *all4613* ilvG, acetolactate synthase I/II/III large subunit [EC:2.2.1.6]	*alr2811* valine- pyruvate aminotransferase [EC:2.6.1.66] *alr3809* carbamoyl-phosphate synthase large subunit [EC:6.3.5.5] *alr4853* aspartate aminotransferase [EC:2.6.1.1] *alr3293* threonine synthase [EC:4.2.3.1]	
Transporters		*alr0140* peptide/nickel transport system substrate- binding protein periplasmic oligopeptide- binding ABC transporter Quorum sensing	*alr1554* ATP-binding cassette, subfamily B		*alr4164* periplasmic amino acid-binding protein of amino acid ABC transporter	*alr3938* ABC transporter iron binding protein high-affinity iron ion transport
Regulatory proteins Signaling		*all4662* cyclic-di-GMP-binding protein		*all0089* Uncharacterized conserved protein YggE, contains kinase-interacting SIMPL domain *all0129* two-component system, OmpR family, response regulator RpaA		
Stress response					*all1541* peroxi- redoxin 2 family protein/ glutaredoxin	
Transcription		*all5263* sigA|RNA polymerase sigma factor RpoD				
Translation				*all4193* small ribosomal protein S13		
Secondary metabolites		*alr0599* 1-deoxy- xylulose 5-phosphate synthase EC:2.2.1.7				
Hypothetical proteins	*alr4505* *all1411* *asl4547* *alr2889* *asr3294*	*all4662*	*alr4505* *all1411* *asr1156*		*alr4505* *alr4504* *all1411* *asl4547*	*all3826*

## Data Availability

The data presented in this study are available at https://doi.org/10.3390/toxins13050325.

## Supplementary Materials

The following are available online at https://www.mdpi.com/article/10.3390/toxins13050325/s1, Figure S1: The scheme represents a protein network of nitrogenase molybdenum-iron protein NifK and its protein partners, Figure S2: The scheme shows the impact of BMAA on four enzymes involved in porphyrin and chlorophyll metabolism in diazotrophically grown cells of *Nostoc* 7120, Figure S3: The protein network of bicarbonate transport system ATP-binding protein cmpD (alr2880) and its protein partners, Figure S4: The scheme shows the impact of BMAA on four enzymes involved in the anabolic pentose phosphate pathway in diazotrophically grown cells of *Nostoc* 7120, Figure S5: The scheme shows the impact of BMAA on the several identified enzymes that are essential for functioning of glycolysis/gluconeogenesis pathways in diazotrophically grown cells of *Nostoc* 7120, Figure S6: The scheme shows the impact of BMAA on the identified enzymes that are involved in the in alanine, aspartate and glutamate metabolism in diazotrophically grown cells of *Nostoc* 7120, Figure S7: The scheme shows a protein network of periplasmic amino acid-binding protein, ABC transporter (alr4164) and its protein partners, Figure S8. This scheme presents a protein network of the RNA-binding protein rbpD (asl4022) and its protein partners, Figure S9: This scheme presents a protein network of ABC transporter iron binding protein (*alr3938*) and its protein partners, Figure S10: This scheme presents a protein network of the starvation-inducible DNA-binding protein (all4145), Figure S11: This scheme presents a protein network of the single-stranded DNA-binding protein (*alr0088*) and its protein partners, Figure S12: This scheme presents a protein network of the hypothetical protein (*alr4505*) and its protein partners, Figure S13: This scheme presents a protein network of the hypothetical protein (*asl4547*) and its protein partners, Figure S14: This scheme presents a protein network of the hypothetical protein (*all1411*) and its protein partners, Figure S15: This scheme presents a protein network of the hypothetical protein (*all3826*) and its protein partners, Table S1: Original proteomic data, Table S2: Gene co-expression data for identified hypothetical proteins in the proteome of diazotrophic *Nostoc* sp. PCC 7120 under BMAA treatment. The data was assembled according to ALCOdbCyano (http://alcodb.jp/cyano/).
