# Supplementary material for: β-N-Methylamino-L-Alanine (BMAA) Causes Severe Stress in Nostoc sp. PCC 7120 Cells under Diazotrophic Conditions: A Proteomic Study
Source: Toxins (Basel). 2021 Apr 30;13(5):325. doi: 10.3390/toxins13050325 (PMC8147232; doi:10.3390/toxins13050325)
Supplement: Supplementary file 1 [file toxins-13-00325-s001.zip › MDPI_Toxins_Supplementary.pdf]

Supplementary Materials: β-N-Methylamino-L-Alanine (BMAA) Causes Severe Stress in *Nostoc* sp. PCC 7120 Cells under Diazotrophic Conditions: a Proteomic Study

Olga A. Koksharova, Ivan O. Butenko, Olga V. Pobeguts, Nina A. Safronova and Vadim M. Govorun

Table S2. Gene coexpression data for some of the identified hypothetical proteins in proteome of diazotrophic *Nostoc* sp. PCC 7120 under BMAA treatment according to ALCOdbCyano (http://alcodb.jp/cyano/) are shown. The coexpressed genes encoding proteins identified in this study are marked by **bold**.

№	Hypothetical Gene	Pair Similar Regulated Proteins (This Study)	List of Co-expressed Genes from the ALCOdbCyano Database
**Up-shifted Hypothetical Proteins**			
1	*alr4505*		alr2991 DnaJ protein
2	*alr4504*		alr4332 Ribonuclease HII
3	*all1411*		all1588 33kD chaperonin, heat shock protein HSP33
4	***all0646***	***all0646***<br>***all0865***	all1221 Biogenesis of thylakoid protein A<br>all0868 Carbon dioxide concentrating mechanism protein CcmK<br>all0867 Carbon dioxide concentrating mechanism protein CcmK<br>**all0865** Carbon dioxide concentrating mechanism protein CcmM<br>all0813 Heterocyst-specific glycolipids-directing protein HglK
5	**all2705**	***all2705***<br>***all3572***	all2846 GMP synthase (glutamine-hydrolyzing)<br>**all3572** Photosystem II protein D1
6	*all0459*		all1173 DNA-binding protein, starvation-inducible<br>all0571 Cyanophycinase
7	***alr2055***	***alr2055***<br>***alr1896***	***alr1896*** Chaperonin GroEL<br>*alr1952* Signal recognition particle protein
8	*alr0114*		*alr1614*Toxin secretion ABC transporter ATP-binding protein
9	*all5218*		*alr4961* DNA repair protein RecN
Down-shifted hypothetical proteins			
1	*all1351*		*all0868* Carbon dioxide concentrating mechanism protein CcmK<br>*all0865* Carbon dioxide concentrating mechanism protein CcmM<br>*all1140* Similar to N-acetylmuramoyl-L-alanine amidase<br>*all1683* Phosphoserine aminotransferase
2	*all3941*		*alr4533* Signal transducer ampG1 homolog<br>*all2584* Ferrichrome ABC transporter, ATP-binding protein<br>*all2586* Iron(III) dicitrate transport system permease protein
3	*all4296*		*alr1978* ABC transporter, ATP-binding protein
4	*alr1850*		*alr4553* Similar to heat shock protein DnaJ<br>*alr3056* Histidinol dehydrogenase; HisD<br>*alr2272*Acyl-[acyl-carrier-protein]-UDP--N-acetylglucosamine o-acyltransferase
5	*all3826*		*all2610* Ferrichrome-iron receptor

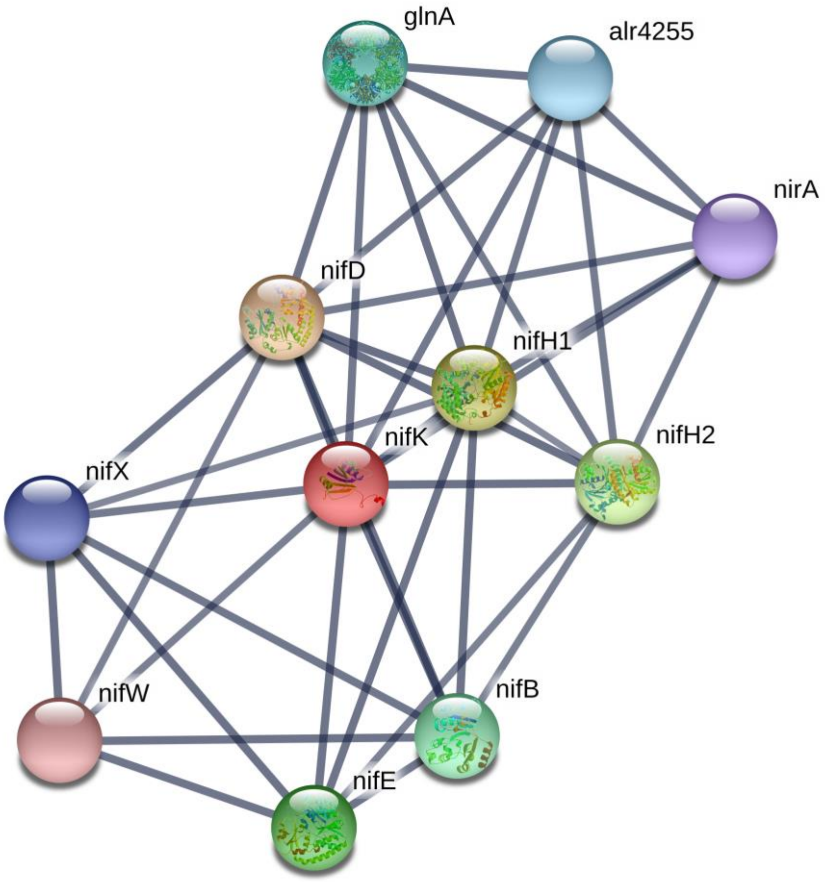

Figure S1. The scheme represents a protein network of nitrogenase molybdenum-iron protein NifK and its protein partners, according to STRING, where nifB, nifD, nifH1, nifH2, nifE, nifX and nifW are nitrogenase structural proteins and/or nitrogenase accessory proteins; glnA is glutamine synthetase; alr4255 is glutamate dehydrogenase; nirA is nitrite reductase.

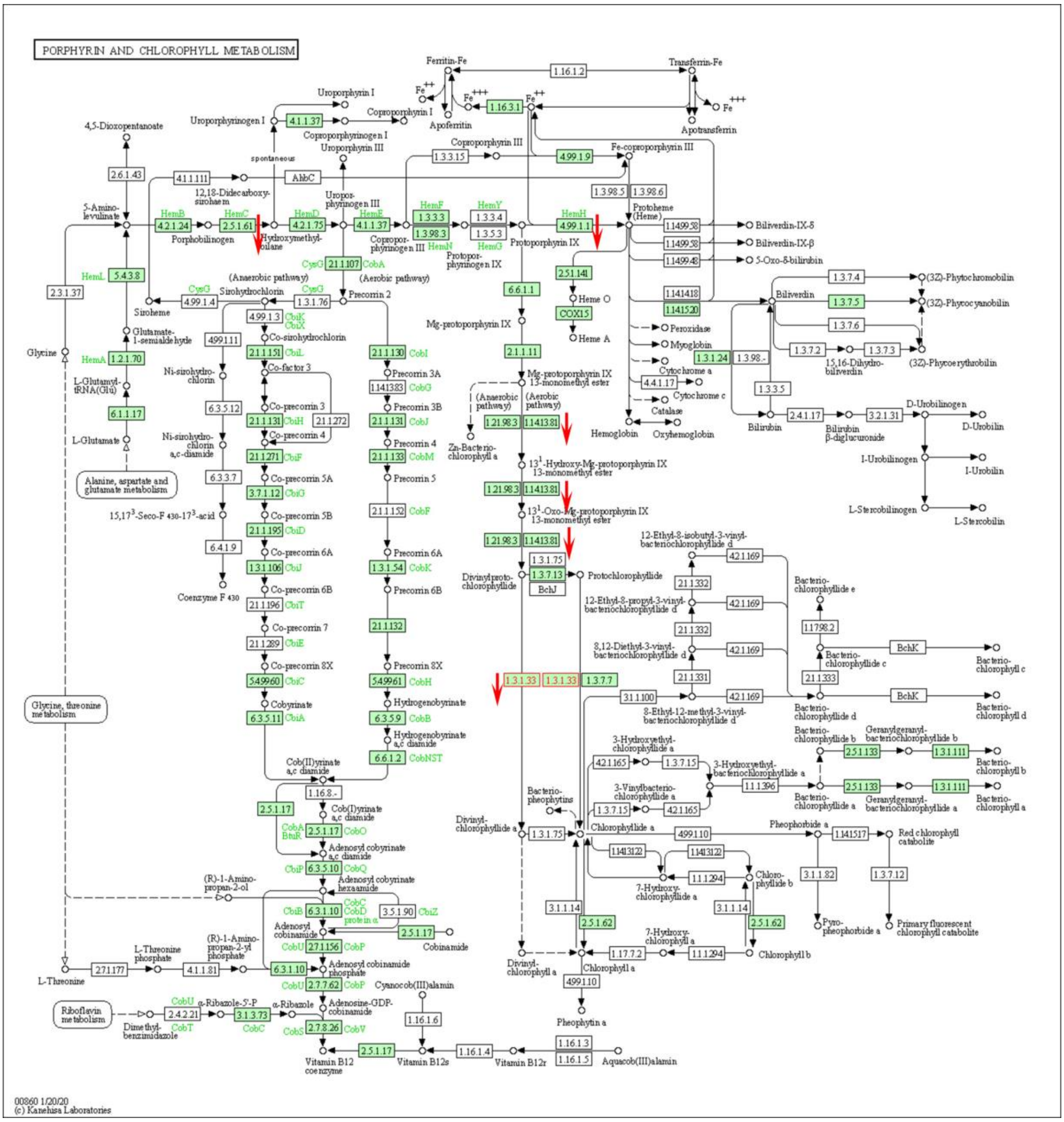

Figure S2. The scheme shows the BMAA impact on four enzymes involved in porphyrin and chlorophyll metabolism in diazotrophically grown cells of *Nostoc* 7120. (https://www.genome.jp/kegg-bin/show_pathway?ana00860+alr1878#). Enzymes the hydroxymethylbilane synthase, hemC [EC:2.5.1.61], the protoporphyrin/coproporphyrin ferrochelatase, hemH [EC:4.99.1.1 4.99.1.9], the protochlorophyllide reductase [EC:1.3.1.33] and the magnesium-protoporphyrin IX monomethyl ester (oxidative) cyclase [EC:1.14.13.81] are downregulated (marked by red arrows).

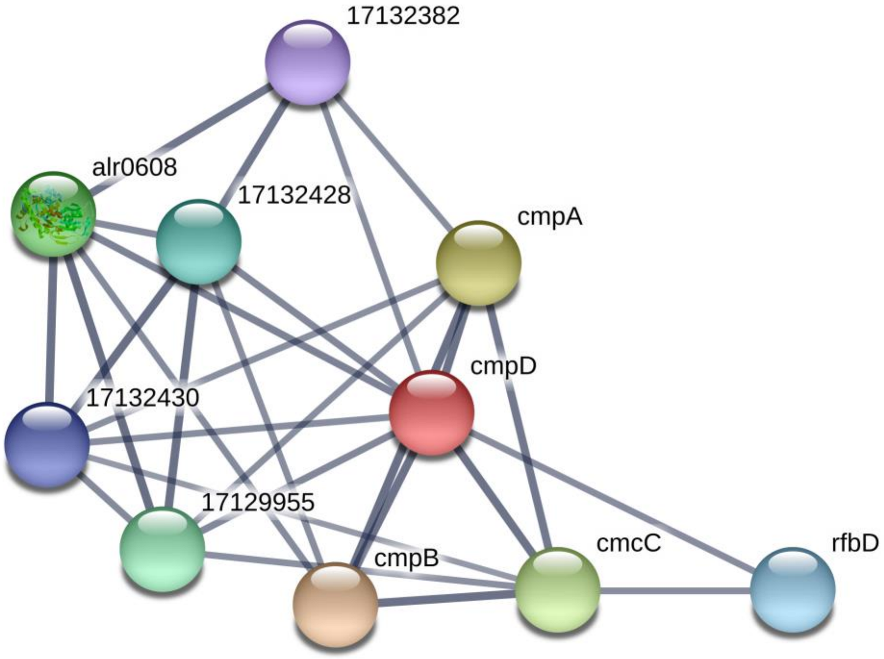

Figure S3. The protein network of bicarbonate transport system ATP-binding protein cmpD (alr2880) and its protein partners, according to STRING. The protein network is represented with the following 9 proteins: cmpA, cmpB, cmpC are proteins that are parts of the ABC transporter complex CmpABCD; rfbD is the dTDP-4-dehydrorhamnose reductase; . alr 0608 is the nitrate transport protein NrtA; proteins 1712955, 17132430, 17132382 are three nitrate transport nrtB proteins; protein 17132428 is the nrtA.

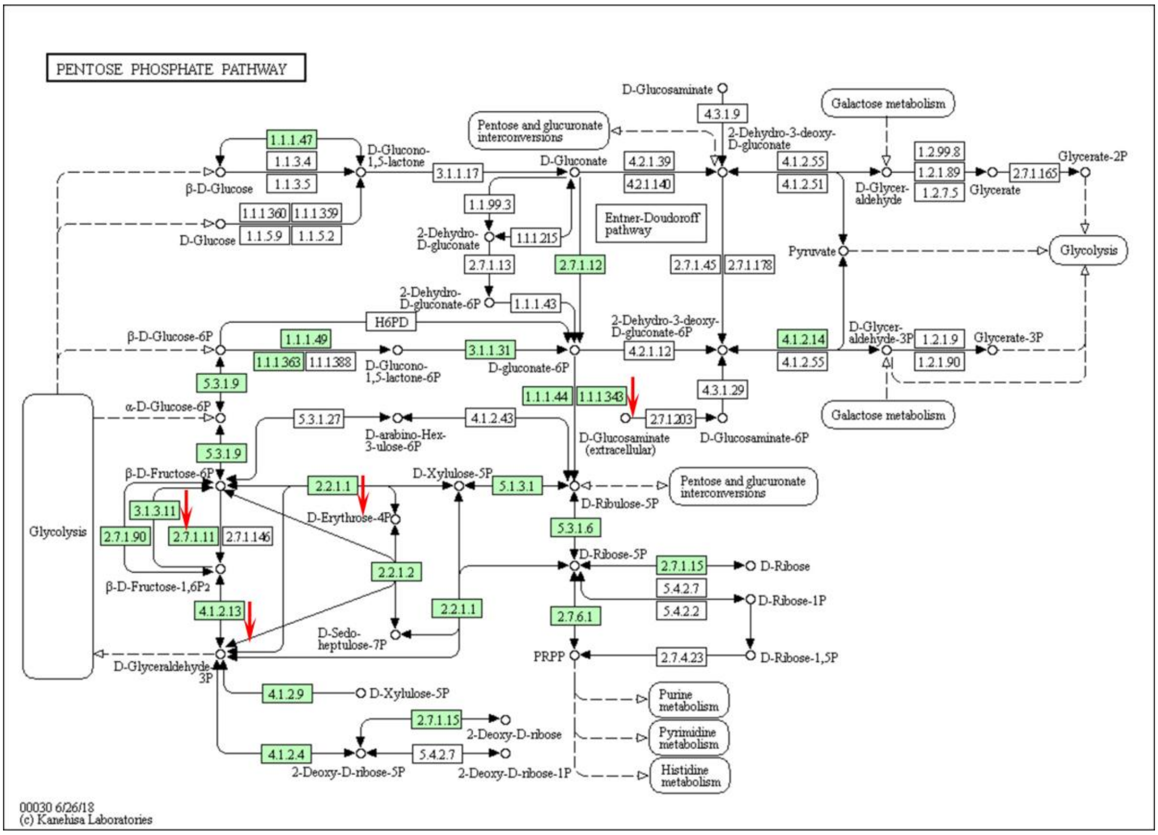

Figure S4. The scheme shows the BMAA impact on four enzymes involved in the anabolic pentose phosphate pathway in diazotrophically grown cells of *Nostoc* 7120. (https://www.genome.jp/kegg-bin/show_pathway?ana00030+alr1041). Four enzymes were downregulated (marked by red arrows) under BMAA treatment (marked by red arrows). Among them were found 6-phosphogluconate dehydrogenase [EC:1.1.1.44 1.1.1.343], *alr5275*; transketolase [EC:2.2.1.1], *alr3344*; Fructose-bisphosphate aldolase, class II EC:4.1.2.13, *all4563* and fructose-1,6-bisphosphatase II/sedoheptulose-1,7-bisphosphatase (FBP/SBPase)[EC:3.1.3.11 3.1.3.37], *alr1041*.

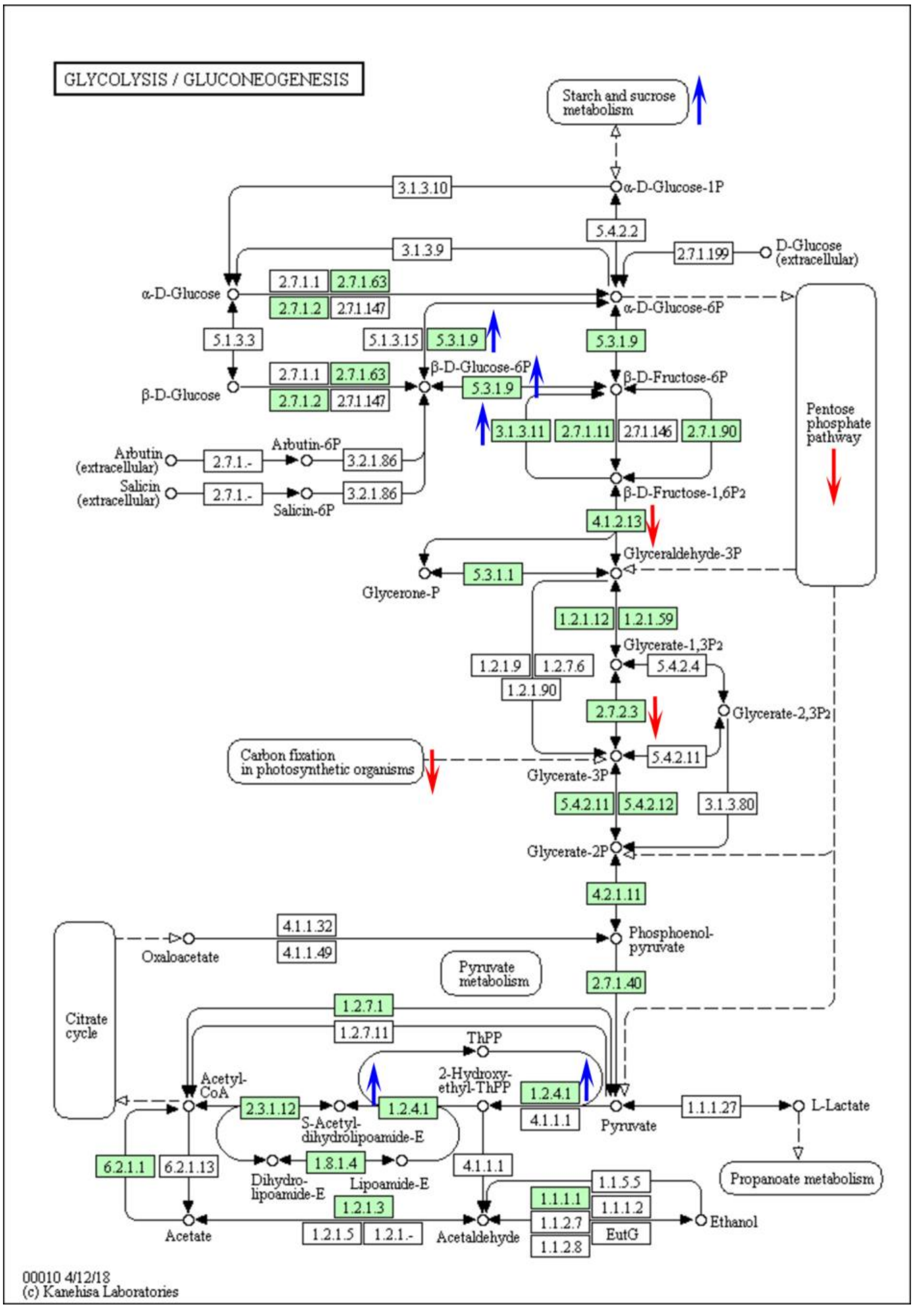

Figure S5. The scheme shows the BMAA impact on the identified enzymes that are involved in the glycolysis/gluconeogenesis pathways in diazotrophically grown cells of *Nostoc* 7120. (https://www.genome.jp/kegg-bin/show_pathway?ana00010+all4131). Two enzymes, Fructose-bisphosphate aldolase, class II EC:4.1.2.13, all4563 and fructose-1,6-bisphosphatase II/sedoheptulose-1,7-bisphosphatase (FBP/SBPase)[EC:3.1.3.11 3.1.3.37], alr1041, are downregulated (marked by red arrows). The other enzymes, fructose-1,6-bisphosphatase I [EC:3.1.3.11], glucose-6-phosphate isomerase [EC:5.3.1.9] and pyruvate dehydrogenase E1 component beta subunit [EC:1.2.4.1], are upregulated (marked by blue arrows).

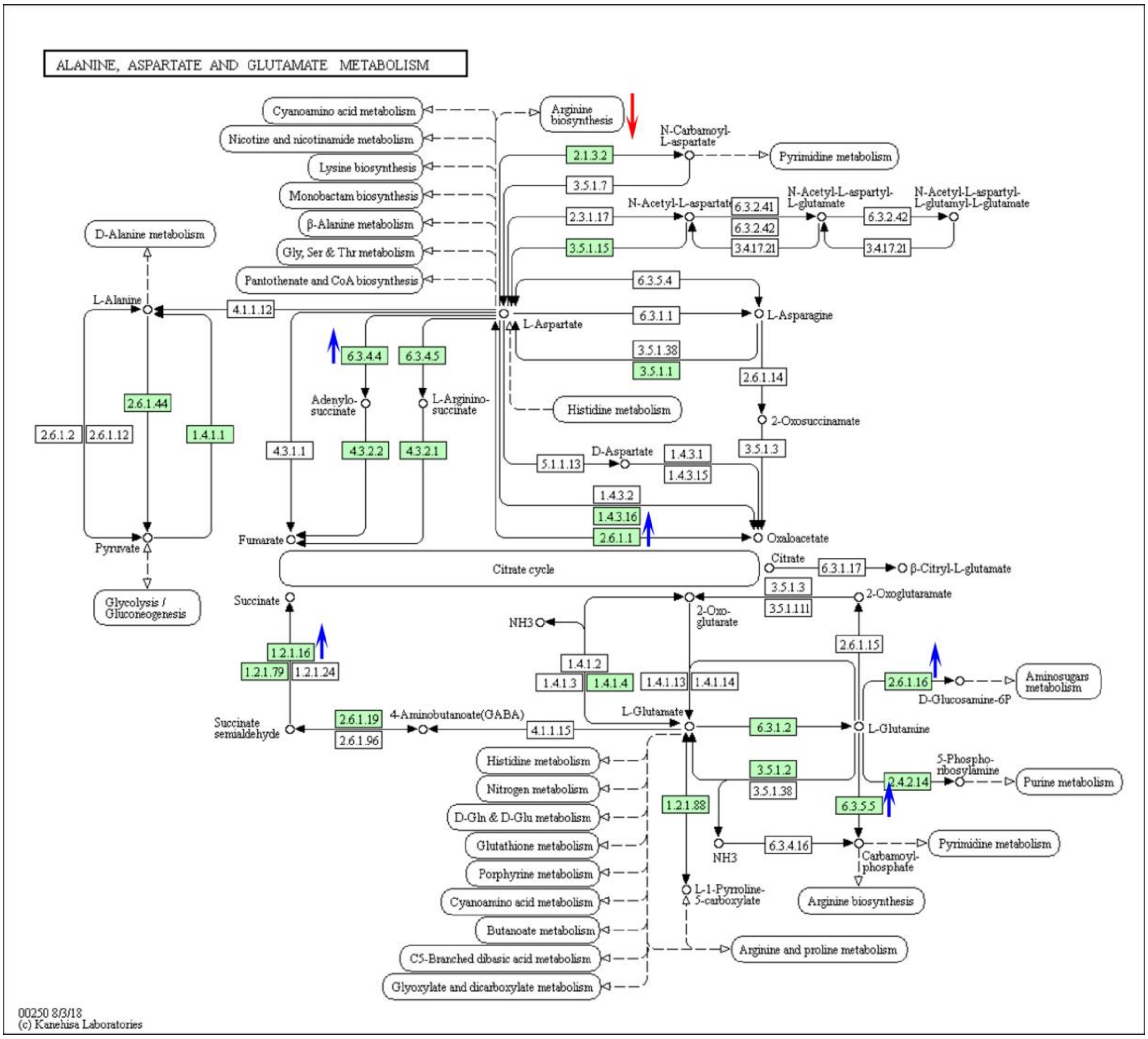

Figure S6. The scheme shows the BMAA impact on the identified enzymes that are involved in the in alanine, aspartate and glutamate metabolism in diazotrophically grown cells of *Nostoc* 7120. (https://www.genome.jp/kegg-bin/show_pathway?ana00250+alr3809). Five enzyme, carbamoyl-phosphate synthase large subunit [EC:6.3.5.5], adenylosuccinate synthase [EC:6.3.4.4], succinate-semialdehyde dehydrogenase/glutarate-semialdehyde dehydrogenase [EC:1.2.1.16 1.2.1.79 1.2.1.20], aspartate aminotransferase [EC:2.6.1.1] and glutamine–fructose-6-phosphate transaminase (isomerizing), nodM, [EC:2.6.1.16], are upregulated (marked by blue arrows).

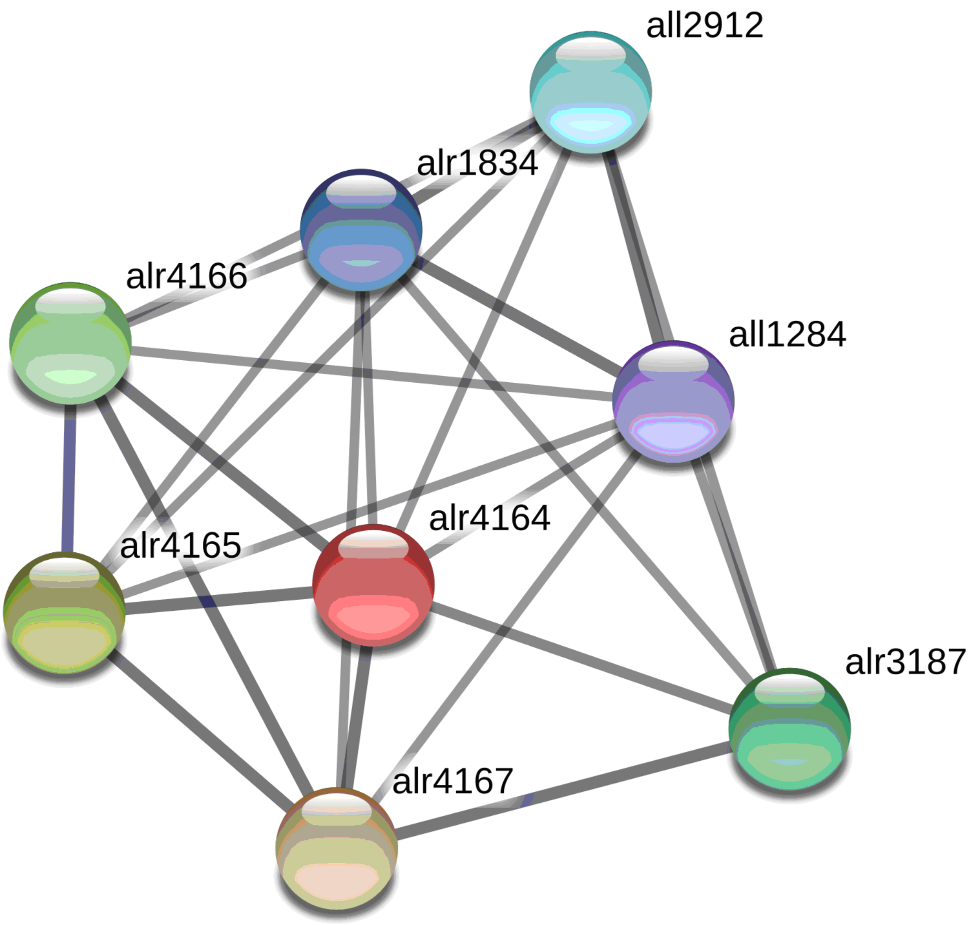

Figure S7. The scheme shows s a protein network of periplasmic amino acid-binding protein, ABC transporter (*alr4164*) (red ball) and its protein partners according to STRING (https://string-db.org). Protein all2912 is ATP-binding protein of branched-chain amino acid ABC transporter; alr1834 is Putative periplasmic branched chain amino acid binding protein ; all1284, alr4166, alr 4165 are Permease protein of amino acid ABC transporters; alr4167 is ATP-binding protein of amino acid ABC transporter; alr3187 is Glutamine-binding periplasmic protein of glutamine ABC transporter.

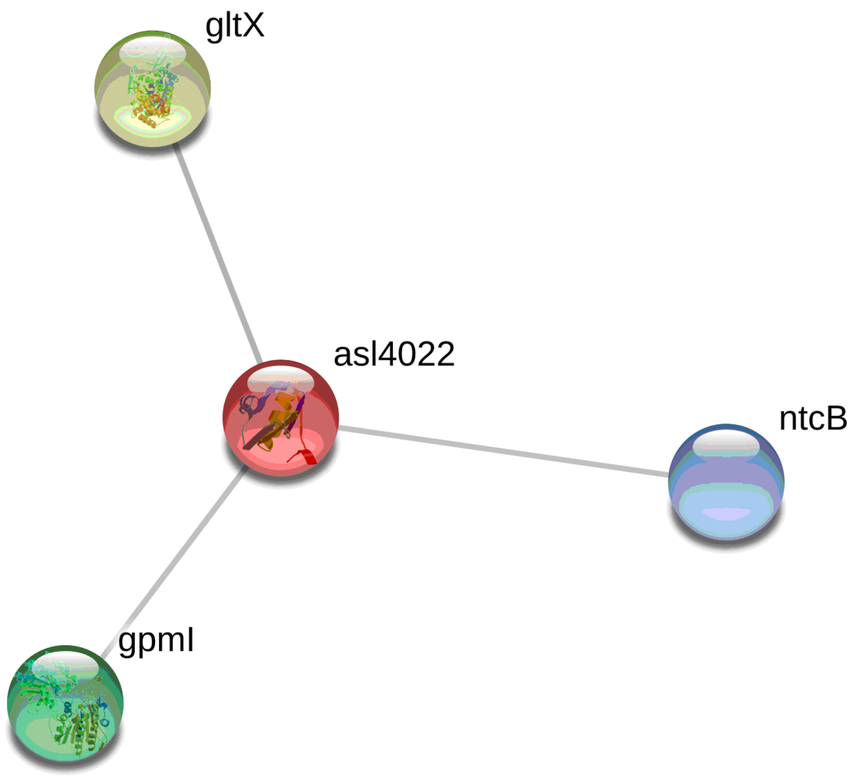

Figure S8. This scheme presents a protein network of the RNA-binding protein rbpD (*asl4022*) and its protein partners, according to the STRING. Protein *rbpD* interacts with gltX (Glutamate--tRNA ligase that catalyzes the attachment of glutamate to tRNA(Glu)) and with ntcB and gpml proteins, where ntcB is a Nitrogen assimilation transcriptional activator (*all0602*) and gpml is a 2,3-bisphosphoglycerate-independent phosphoglycerate mutase (*all4182*).

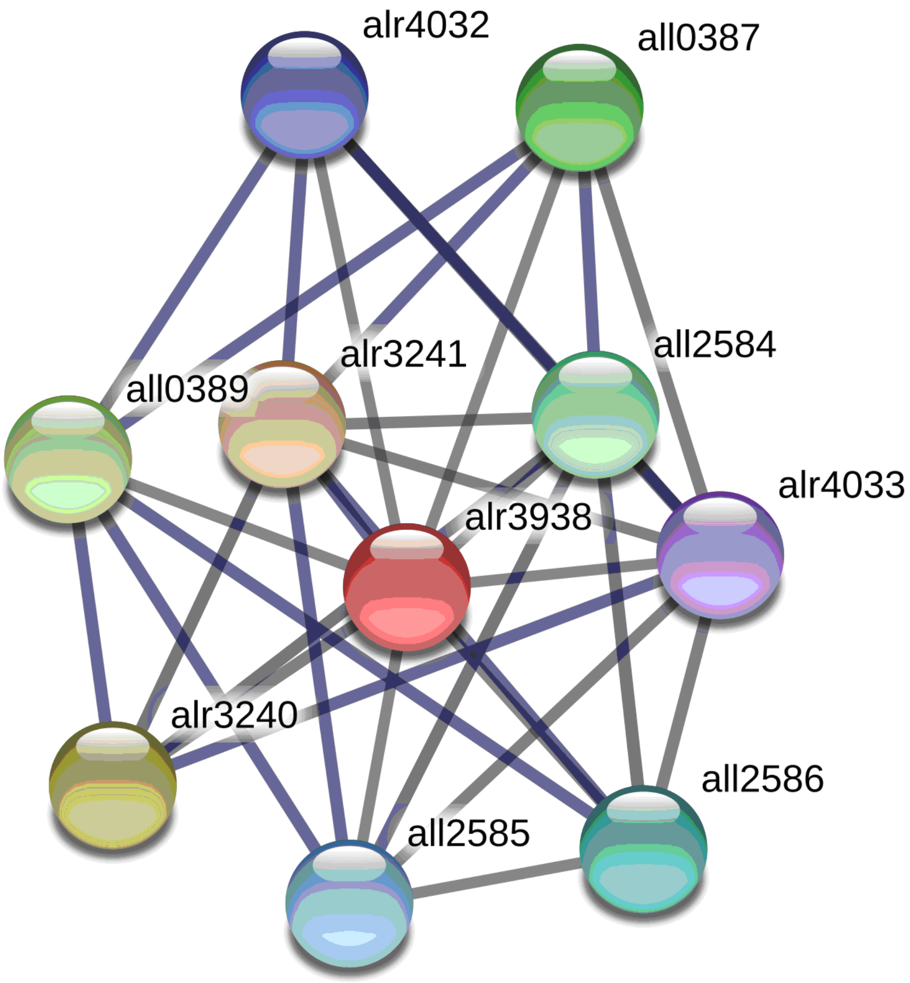

Figure S9. This scheme presents a protein network of ABC transporter iron binding protein (*alr3938*) (red ball) and its protein partners according to STRING (https://string-db.org). Protein *alr4032* is permease protein of iron(III) ABC transporter; protein (*all0387*) is permease protein of iron(III) ABC transporter; *all2584* is ATP-binding protein of ferrichrome ABC transporter; *alr4033* is ATP-binding protein of ferrichrome ABC transporter; *all2585* is Iron(III) dicitrate transport system permease protein; *all2586* is Iron(III) dicitrate transport system permease protein; *all0389* is ATP-binding protein of iron(III) ABC transporter; protein (*alr3240*) is permease protein of ferrichrome ABC transporter;*alr3241* is ATP-binding protein of ferrichrome ABC transport;.

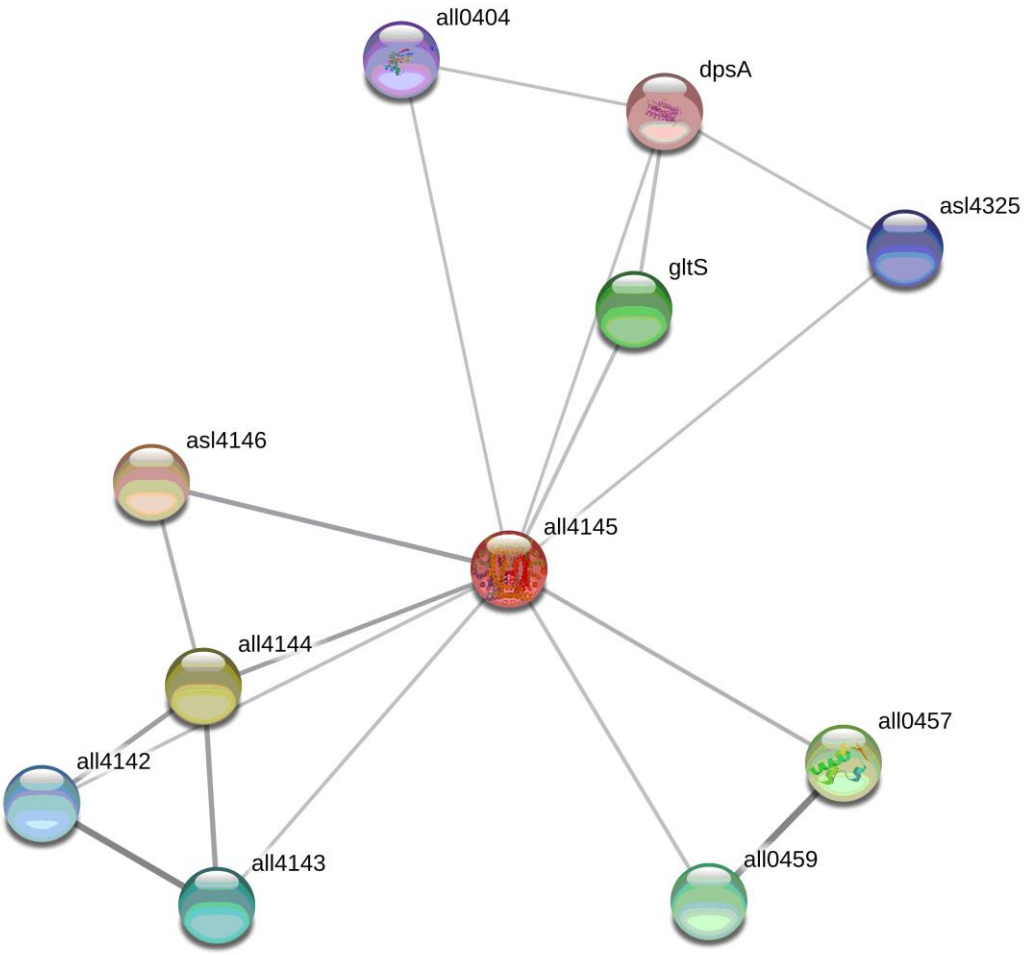

Figure S10. This scheme presents a protein network of the starvation-inducible DNA-binding protein (all4145) (red ball) and its protein partners, according to STRING. The protein network is represented with the following 10 protein partners: dpsA (alr3808) is a nutrient stress-induced DNA-binding protein;. gltS (alr4344) is a ferredoxin-glutamate synthase;. all0457 and all0459 are uncharacterized low temperature-induced proteins;. all4142 and all4143 are unknown proteins; all4144 is probably a chaperon;asl4146 is a hypothetical protein;. all0404 and asl4325 represent ClpS adapter protein of ATP-dependent Clp protease.

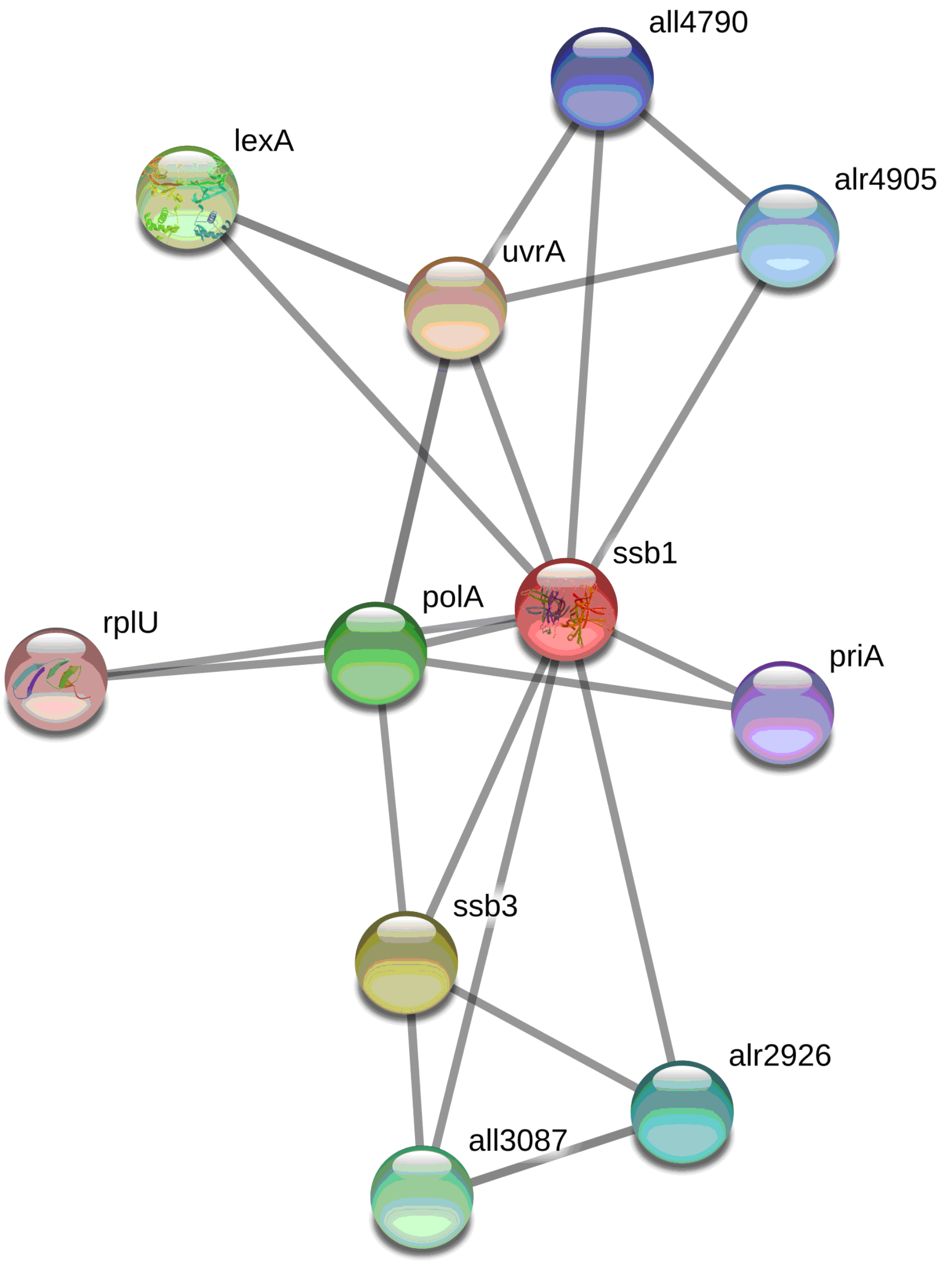

Figure S11. This scheme presents a protein network of the single-stranded DNA-binding protein (*alr0088*) (red ball) and its protein partners according to STRING (https://string-db.org). Protein lexA is repressor; that represses a number of genes involved in the response to DNA damage (SOS response), including recA and lexA; protein UvrA is an ATPase and a DNA-binding protein that is part of the UvrABC repair system catalyzes the recognition and processing of DNA lesions; all4790 and alr2926 are uncharacterized proteins; alr4905 is two-component sensor histidine kinase; rplU is 50S ribosomal protein L21; this protein binds to 23S rRNA in the presence of protein L20; polA is DNA polymerase I that in addition to polymerase activity, exhibits 5′-3′ exonuclease activity; ssb3 (*all4779*) is single-strand DNA-binding protein that has 100% identity with temperature-regulated DNA-binding protein of *Anabaena variabilis* ; all3087 is calcium and DNA-binding protein; protein priA is the primosomal protein N′, which involved in the restart of stalled replication forks.

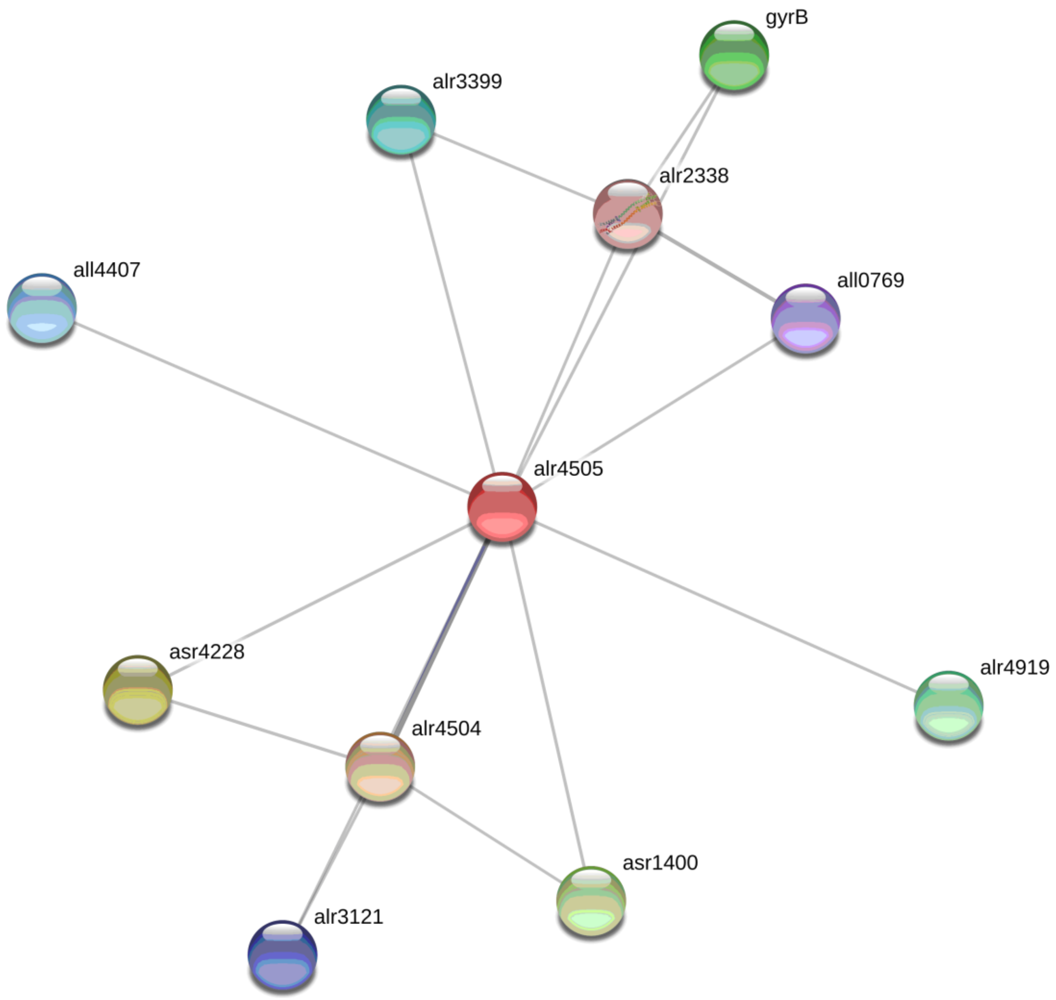

Figure S12. This scheme presents a protein network of the hypothetical protein (*alr4505*) (red ball) and its protein partners according to STRING (https://string-db.org). (1) Protein gyrB is DNA gyrase subunit B that is a type II topoisomerase that negatively supercoils closed circular double-stranded (ds) DNA in an ATP-dependent manner to modulate DNA topology and maintain chromosomes in an underwound state. Negative supercoiling favors strand separation, and DNA replication, transcription, recombination and repair, all of which involve strand separation. Also this protein is able to catalyze the interconversion of other topological isomers of dsDNA rings, including catenanes and knotted rings. Type II topoisomerases break and join 2 DNA strands simultaneously in an ATP-dependent manner. (2) Protein alr3399 is segregation and condensation protein A that participates in chromosomal partition during cell division and may act via the formation of a condensin-like complex containing Smc and ScpB that pull DNA away from mid-cell into both cell halves; (3) alr2338 is integral membrane protein with unknown function; (4) all0769 is Acetyl-CoA synthetase; (5) all4407 and (6) asr1400 are uncharacterized proteins; (7) alr4919 is AAA protein (ATPases Associated with diverse cellular Activities) that can be involved in processes such as DNA replication, protein degradation, membrane fusion, microtubule severing, peroxisome biogenesis, signal transduction and the regulation of gene expression; (8) asr4228 is protein with unknown function; (9) alr4504 is hypothetical protein; (10) alr3121 is two-component hybrid sensor and regulator.

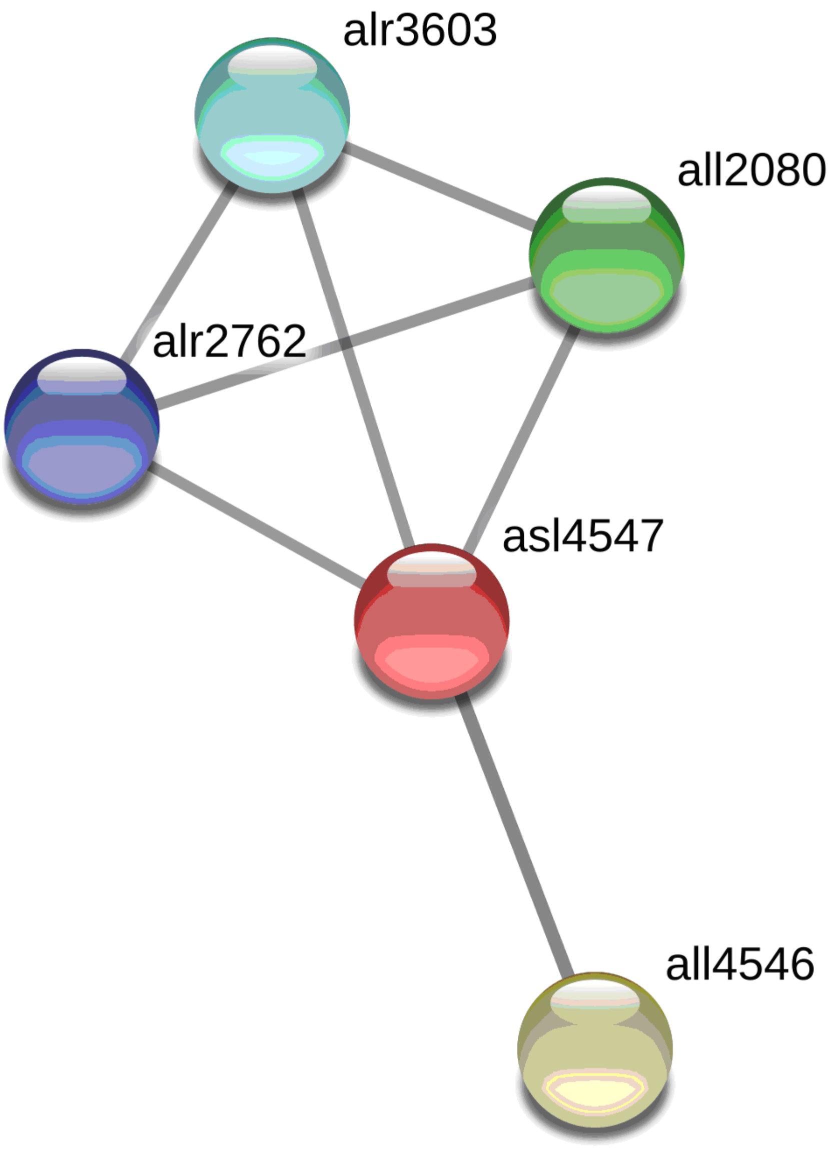

Figure S13. This scheme presents a protein network of the hypothetical protein (*asl4547*) (red ball) and its protein partners according to STRING (https://string-db.org). Protein all4546 is ABC transporter ATP-binding protein; alr2762 is transmembrane uncharacterized protein; alr3603 is hypothetical protein with exonuclease VII domain; all2080 is an AbrB family transcriptional regulator.

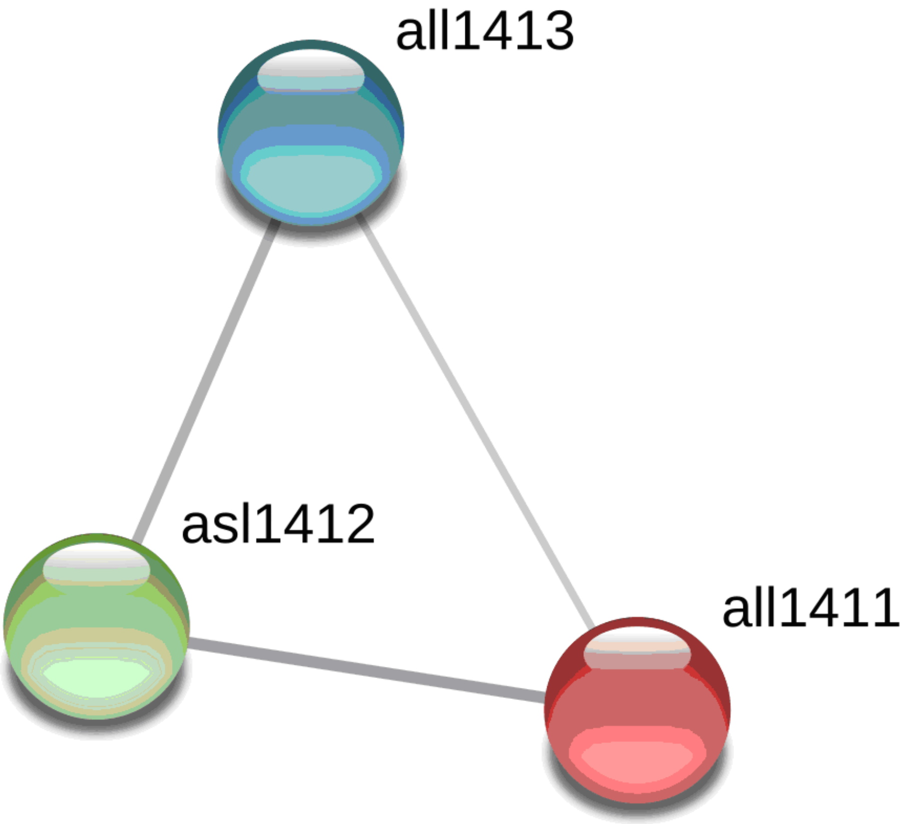

Figure S14. This scheme presents a protein network of the hypothetical protein (*all1411*) (red ball) and its protein partners according to STRING (https://string-db.org). Protein asl1412 is uncharacterized protein; all1413 is uncharacterized membrane protein.

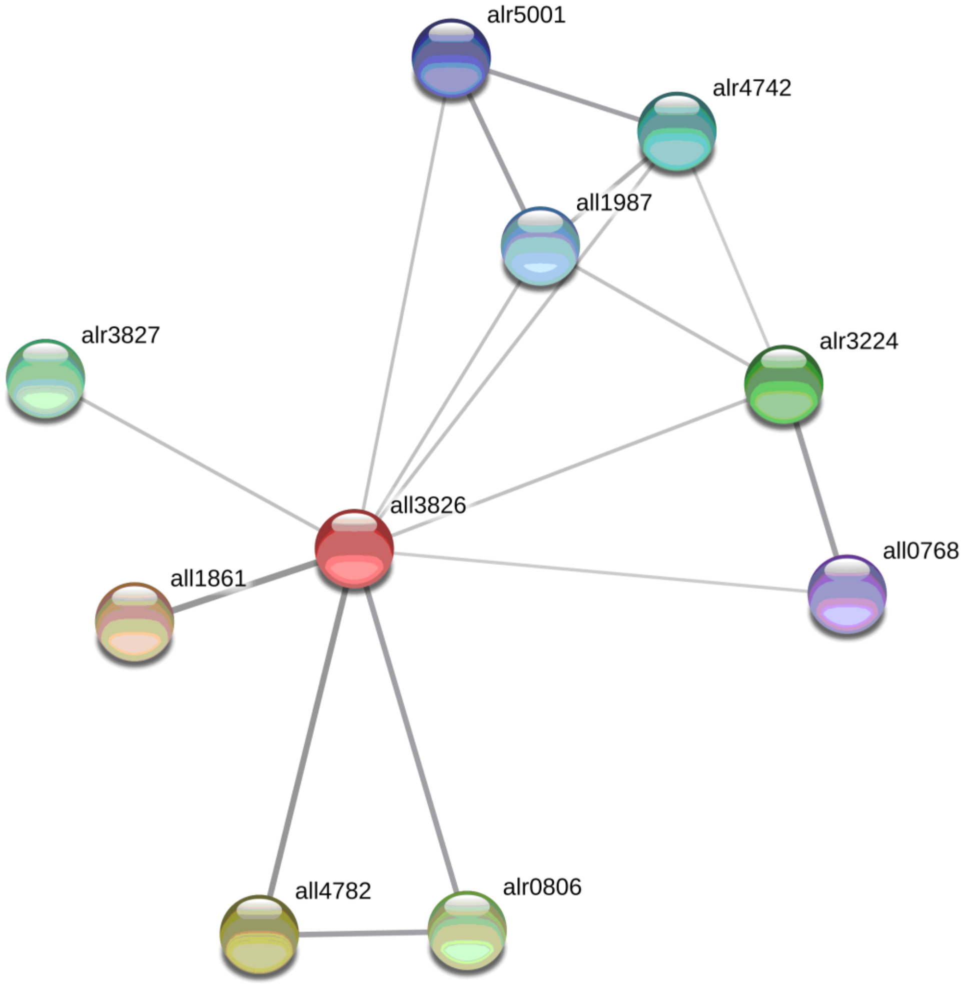

Figure S15. This scheme presents a protein network of the hypothetical protein (all3826) (red ball) and its protein partners according to STRING (https://string-db.org). Proteins alr5001, alr3224, all4782, all1987 and alr0806 are uncharacterized protein; alr4742 and alr3827 are membrane proteins; all0768 is N-acetyltransferase domain-containing protein; all1861 is protein with Peptidoglycan-bd-like domain.
